# Outcomes of a Geriatric Liaison Intervention to Prevent the Development of Postoperative Delirium in Frail Elderly Cancer Patients: Report on a Multicentre, Randomized, Controlled Trial

**DOI:** 10.1371/journal.pone.0064834

**Published:** 2013-06-19

**Authors:** Liesbeth Hempenius, Joris P. J. Slaets, Dieneke van Asselt, Geertruida H. de Bock, Theo Wiggers, Barbara L. van Leeuwen

**Affiliations:** 1 University Center for Geriatric Medicine, University Medical Center Groningen, University of Groningen, Groningen, The Netherlands; 2 Geriatric Center, Medical Center Leeuwarden, Leeuwarden, The Netherlands; 3 Department of Epidemiology, University Medical Center Groningen, University of Groningen, Groningen, The Netherlands; 4 Department of Surgery, University Medical Center Groningen, University of Groningen, Groningen, T`he Netherlands; Cardiff University, United Kingdom

## Abstract

**Background:**

Delirium is a serious and common postoperative complication, especially in frail elderly patients. The aim of this study was to evaluate the effect of a geriatric liaison intervention in comparison with standard care on the incidence of postoperative delirium in frail elderly cancer patients treated with an elective surgical procedure for a solid tumour.

**Methods:**

Patients over 65 years of age who were undergoing elective surgery for a solid tumour were recruited to a multicentre, prospective, randomized, controlled trial. The patients were randomized to standard treatment versus a geriatric liaison intervention. The intervention consisted of a preoperative geriatric consultation, an individual treatment plan targeted at risk factors for delirium, daily visits by a geriatric nurse during the hospital stay and advice on managing any problems encountered. The primary outcome was the incidence of postoperative delirium. The secondary outcome measures were the severity of delirium, length of hospital stay, complications, mortality, care dependency, quality of life, return to an independent preoperative living situation and additional care at home.

**Results:**

In total, the data of 260 patients were analysed. Delirium occurred in 31 patients (11.9%), and there was no significant difference between the incidence of delirium in the intervention group and the usual-care group (9.4% vs. 14.3%, OR: 0.63, 95% CI: 0.29–1.35).

**Conclusions:**

Within this study, a geriatric liaison intervention based on frailty for the prevention of postoperative delirium in frail elderly cancer patients undergoing elective surgery for a solid tumour has not proven to be effective.

**Trial Registration:**

Nederlands Trial Register Trial ID NTR 823

## Introduction

The world's population is ageing, and it is predicted that when this ageing reaches its peak in 2050, 27.6% of Europeans will be over 65 years of age [1]. As the population ages, the prevalence of illness and hospitalization increases. Before long, cancer will be the leading cause of death, and more than half of new solid tumours will occur in patients over 70 years of age [2]. Surgery is an essential part of the multimodal treatment of solid tumours, and frail elderly patients are especially at risk of developing postoperative complications [3]–[5].

Postoperative delirium is a common and serious complication in hospitalized elderly people. Its incidence varies from less than 10% to 50% after orthopaedic [6], abdominal [7]–[11] and cardiac surgery [12]. Delirium is associated with persistent functional and cognitive decline, increased morbidity and mortality, longer hospital stays, higher rates of nursing home placement and increased health-care costs [13]–[17]. Mortality rates vary from 4% to 20% in patients who develop delirium during their hospital stay [7], [18]. It is therefore important to optimize the care for this growing group of patients.

The current treatment to prevent delirium consists of pharmacological and non-pharmacological, mostly multicomponent, interventions. Both have proven effective [19], [20], but until now most delirium prevention studies of the elderly included orthopaedic patients (usually hip-fracture patients) or patients from an acute care unit.

The aim of this multicentre, randomized, clinical trial was to evaluate the effect of a geriatric liaison intervention in comparison with the effect of standard care on the incidence of postoperative delirium in frail elderly cancer patients treated with an elective surgical procedure for a solid tumour.

## Methods

The protocol for this trial and supporting CONSORT checklist are available as supporting information; see Checklist S1 and Protocol S1.

### Ethics statement

The study was approved by the Medical Ethical Committee of the University Medical Center Groningen, trial ID NTR 823.

### Study design

The study, entitled Liaison Intervention in Frail Elderly (LIFE), was a multicentre, randomized clinical trial. The participating centres were the University Medical Center Groningen (serving a population of three million people), the Medical Center Leeuwarden (a large teaching hospital) and Diaconessenhuis Leiden (a community hospital). All participating centres are located in the Netherlands.

### Participants

From June 2007 to June 2010 all consecutive patients over 65 years of age undergoing elective surgery for a solid tumour were assessed with the Groningen Frailty Indicator (GFI) [21] at the outpatient departments of general surgery, gynaecology, ear, nose and throat medicine and maxillofacial surgery at the participating centres. The GFI is a short 15-item screening instrument used to determine an individual's level of frailty. It screens for the loss of function and resources in four domains of functioning: physical (mobility functions, multiple health problems, physical fatigue, vision and hearing), cognitive (cognitive functioning), social (emotional isolation) and psychological (depressed mood and feelings of anxiety). It is an internally consistent scale (Cronbach's Alpha 0.77) [22]. Patients with a GFI score greater than 3 were regarded as frail [21], [22] and recruited to this study. The GFI has not been specifically validated in a cancer population before. After informed consent, the participants were randomly allocated to either the control group or the geriatric liaison intervention group. The randomization was stratified by tumour type. A distinction was made between tumours in the chest or abdomen and tumours elsewhere. The research nurses used an interactive voice response telephone service provided by the University Medical Center Groningen for the randomization.

If it was obvious that patients would be unable to complete the study protocol and follow-up schedule before inclusion, they were excluded from participation (e.g. for logistical reasons or if any extra hospital visits would be too burdensome). Patients unable to fill in the questionnaires used in this study were also excluded.

### Intervention

The multicomponent intervention focused on best supportive care and the prevention of delirium. Patients in the intervention group were assessed preoperatively by a geriatric team and monitored during their hospital stay. As the three participating centres are heterogeneous and this could cause variance in how the intervention was conducted, checklists were used to standardize the intervention as much as possible.

The geriatric team was supervised by a geriatrician, and helped devise the individual care plan. The preoperative comprehensive geriatric assessment by a geriatrician consisted of a medical history, physical examination and follow-up examinations on indication. In order to standardize this consultation a checklist was composed based on expert opinion. This checklist contained items concerning medication, co-morbidities, loss of vision and hearing, nutrition, mobility, depression, incontinence and cognitive, social and instrumental functioning (instrumental Activities of Daily Life ([i]ADL)). An individual treatment plan was drawn up paying specific attention to patient-related risk factors for delirium, namely, cognitive impairment, visual impairment, hearing impairment, malnutrition and impaired mobility. Preventive pharmacological measures were an optional but non-imperative part of the intervention protocol.

During their hospital stay, the patients in the intervention group were assessed daily by a geriatric nurse. A daily checklist was used to ensure the uniformity of the geriatric intervention in the participating centres [23] (Appendix 1). This checklist consisted of nine items: orientation, mobility, anxiety, senses, pain, sleep, intake, defecation and infection. If a problem concerning one of these was encountered, the geriatric nurse or geriatrician contacted the treatment team to discuss the proposed intervention and establish a treatment plan, checking daily to determine whether the advice had been followed.

### Standard care

Patients in the usual-care group received standard care, which means that additional geriatric care was only provided at the request of the treating physician.

### Surgical procedure

Surgical procedures were divided into three categories: minor, intermediate and major according to the duration of the operation and the localization of the tumour (intracavitary versus superficial) (Table 1).

**Table 1:** Classification of the type of surgery by duration of the procedure and tumour localization.

Surgery load	Tumour localization
Minor	Breast and skin
Intermediate	Vulva, cervix, endometrium, uterus, head/neck and retroperitoneum
Major	Gastrointestinal, liver, pancreas, lung, ovary, oropharynx, larynx and intra-abdominal sarcoma

### Outcomes

The primary outcome was the incidence of delirium up to 10 days postoperatively.

Secondary outcome variables were the severity of delirium, length of hospital stay, complications, mortality, care dependency, quality of life, return to an independent preoperative living situation and additional care at home.

### Assessments

The data were collected at admission, during hospital stay and at discharge, using a paper-based standardized form and then entered into Oracle Clinical© Remote Data Capture program by trained research nurses. After entry, the data were checked by an independent individual. The research nurse helped the patients fill in the questionnaires during an interview. See Table 2 for an overview of the assessments.

**Table 2:** Overview of assessments used in the LIFE study.

Time point	Outcome	Scale/measurement used
Selection	Frailty	Groningen Frailty Indicator (GFI)^22^
Baseline	Demographic data	Age, sex, comorbidities, living situation, supportive care, type of surgery
	Quality of Life	Short Form – 36 (SF-36)
	Care dependency	Care Dependency Scale (CDS)
	Cognitive functioning	Mini-Mental State Examination (MMSE)
1st to 10th postoperative day	Sign of delirium	Delirium Observation Scale (DOS) three times a day
	Delirium	Confirm diagnosis by geriatrician or psychiatrist according to DSM IV criteria
	Delirium severity	Delirium Rating Scale – Revised – 98 (DRS-R-98)
	Postoperative complications	
At discharge	Quality of Life	Short Form – 36 (SF-36)
	Care Dependency	Care Dependency Scale (CDS)
	Living situation	
	Supportive care	

The baseline assessment was completed by the research nurses at least 24 hours before surgery and was taken prior to randomization. The baseline assessment included the collection of demographic data; assessment of the quality of life, measured by a Short Form-36 (SF-36) score [24]; care dependency, measured by the Care Dependency Scale (CDS) [25]; and cognitive functioning, measured by the Mini-Mental State Examination (MMSE) [26].

The Delirium Observation Scale (DOS) was used in both groups to screen for delirium. The DOS [27] was recorded three times a day (up to 10 days postoperatively) during the hospital stay by the nurses on the wards to monitor early warning signs of delirium. All nurses on the participating wards were trained by the research nurse to score the DOS. In the case of a mean DOS score ≥3 (possible delirium) a geriatrician or psychiatrist examined the patient to confirm the diagnosis according to the criteria of the *Diagnostic and Statistical Manual of Mental Disorders, Fourth Edition* (DSM IV). The severity of delirium as measured by the highest value of the Delirium Rating Scale – Revised – 98 (DRS-R-98) [28].

The research and ward nurses were not blinded to the group the patients had been assigned to. The doctor diagnosing a possible delirium was, however, masked to the study group.

### Statistical Analysis

To achieve a power of 80% with an α of 5% (one-sided), a β of 95% and an expected drop-out rate of 10%, it was calculated that a total of at least 294 patients would need to be included in this study. The reported incidence of postoperative delirium varies widely from less than 10% to 50%. Based on these data and the fact that this study included a high-risk population, a delirium incidence of 30% was expected in the study population. An absolute reduction of 15% was expected in the intervention group based on Inouye's results (1999) [29].

Differences in baseline characteristics between the groups were examined using a Fisher exact test for nominal variables and a two-sample Smirnov test for ordinal or continuous variables.

For the primary analysis of the effectiveness of the intervention, delirium was considered a binary outcome (present or absent), according to its earliest occurrence, and only one episode of delirium per patient was counted. Univariate binary logistic regression analysis was used and Odds Ratios (ORs) with a 95% Confidence Interval (CI) were calculated to examine the effectiveness of the intervention strategy on the primary and secondary outcomes.

All of the statistical tests were one-sided, with α = 0.05 as the criterion of statistical significance. Furthermore, the analyses were carried out using IBM SPSS Statistics Version 20.

## Results

**Figure 1:**
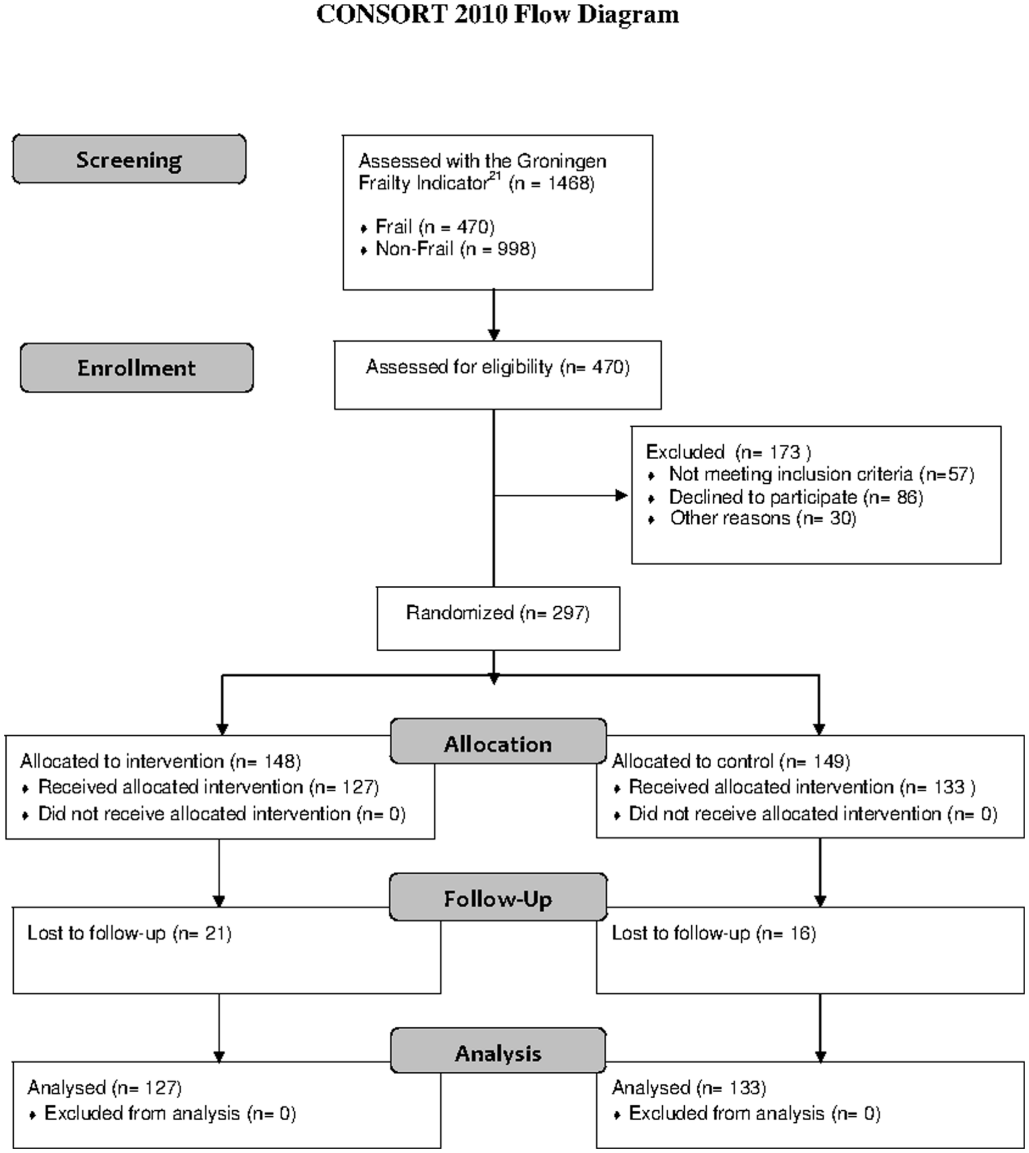
CONSORT diagram for the study.

1468 patients were screened from June 2007 to June 2010 (Figure 1). Of these patients, 470 were found to be frail and 998 non-frail. One hundred and seventy-three frail patients were excluded from the analysis: 57 patients failed to meet the inclusion criteria, 86 refused to participate, 13 were excluded for logistical reasons and 17 patients for reasons unknown. Thirty-seven patients (12.5%) were lost to follow-up: 23 patients were inoperable or were operated on under local anaesthesia, four were lost for logistical reasons, six withdrew informed consent, two died before surgery, one had a benign tumour and one had severe cognitive impairment that was incompatible with the study design. The complete case analysis included 260 patients.

### Baseline measurements

Table 3 shows the characteristics of the patients at the time of inclusion. There were no significant differences between the groups at baseline.

**Table 3:** Baseline characteristics of the patients at inclusion according to study group.

Characteristic	Intervention group (n = 148)	Usual-care group (n = 149)	P-value
Age (years), mean (SD)	77.45 (6.72)	77.63 (7.69)	0.64†
Female, n (%)	92 (62.2%)	98 (65.8%)	0.55‡
**Type of surgery** ∫ **, n (%)**			0.47‡
minor	40 (27.0)	37 (24.8)	
intermediate	28 (18.9)	37 (24.8)	
major	80 (54.1)	75 (50.3)	
**Comorbidities** * **, n (%)**			0.49‡
≤2	57 (39.6)	59 (40.4)	
>2	87 (60.4)	87 (59.6)	
missing	4	3	
**Living situation, n (%)**			0.06‡
independent	125 (87.4)	116 (80.0)	
*alone*	59 (41.3)	55 (37.9)	
*with others*	66 (46.1)	61 (42.1)	
dependent	18 (12.6)	29 (20.0)	
*protected housing*	1 (0.7)	4 (2.8)	
*home for the elderly*	14 (9.8)	22 (15.2)	
*nursing home*	3 (2.1)	3 (2.1)	
missing	5	4	
**Supportive care, n (%)**			
Domestic help			0.45‡
*No*	65 (45.8)	64 (44.4)	
*Yes*	77 (54.2)	80 (55.6)	
Care assistance			0.42‡
*No*	96 (67.6)	100 (69.4)	
*Yes*	46 (32.4)	44 (30.6)	
Informal care			0.41‡
*No*	75 (52.8)	73 (50.7)	
*Yes*	67 (47.2)	71 (49.3)	
Missing	6	5	
**Care Dependency Score, mean (SD)**	72.29 (8.92)	73.53 (9.08)	0.28†
Missing	6	5	
**Mini Mental State Examination, mean (SD)**	26.68 (2.97)	26.33 (3.91)	0.49†
Missing	30	37	
**Short Form-36, mean (SD)**			
Physical Function	46.01 (30.56)	50.03 (30.51)	0.47†
Social Function	67.96 (29.49)	68.36 (27.17)	0.99†
Role Physical	45.08 (34.06)	45.65 (32.55)	0.99†
Role Emotional	62.26 (31.99)	65.46 (30.98)	0.98†
Mental Health	56.99 (18.28)	58.12 (17.15)	1.00†
Vitality	48.91 (20.03)	51.28 (18.55)	0.99†
Bodily Pain	67.86 (29.81)	70.62 (27.07)	0.84†
General Health	45.98 (20.16)	48.05 (18.65)	0.17†
Health Change	30.63 (24.98)	31.55 (25.86)	0.98†
Missing	6, 1 incomplete	4	

† Kolmogorov-Smirnov test,

‡ Fisher's exact test,

∫ Surgery load: Major = gastrointestinal, liver, pancreas, lung, ovary, oropharynx, larynx and intra-abdominal sarcoma. Intermediate = vulva, cervix, endometrium, uterus, head/neck and retroperitoneum. Minor = breast and skin.

* Comorbidities = diabetes, COPD, hypertension, myocardial infarction, other cardiovascular disorders, neurological disorders, cerebrovascular disorders, hearing and vision problems, memory problems in daily life, psychiatric disorders or musculoskeletal disorders.

### Outcomes

The results of the logistic regression analyses for delirium and the secondary outcomes are shown in Tables 4 and 5 (quality of life). Each outcome is discussed separately below.

**Table 4:** Logistic regression analyses (intervention group versus control group).

Outcome	Intervention group n = 127	Control group n = 133	OR (95% CI)
**Primary outcome**			
Delirium, n (%)			
Yes	12 (9.4)	19 (14.3)	0.63 (0.29–1.35)
No	115 (90.6)	114 (85.7)	
*Severity of delirium, median (range)*	*9 (3–30)*	*15 (5–29)*	*p = 0.23*
**Secondary outcomes**			
Complications, n (%)			
>1	42 (33.1)	38 (28.6)	1.24 (0.73–2.10)
≤1	85 (66.9)	95 (71.4)	
Mortality, n (%)			
Yes	10 (7.9)	4 (3.0)	2.76 (0.84–9.03)
No	117 (92.1)	129 (97.0)	
Length of hospital stay (days), n (%)			
Above median	63 (49.6)	57 (42.9)	1.28 (0.77–2.12)
Below median	57 (44.9)	66 (49.6)	
Care dependency*, n (%)			
Increased	86 (74.1)	96 (75.6)	0.93 (0.52–1.65)
Same/decreased	30 (25.9)	31 (24.4)	
Return to independent preoperative living situation, n (%)			
No	37 (32.7)	23 (20.9)	1.84 (1.01–3.37)
Yes	76 (67.3)	87 (79.1)	
Supportive care, n (%)			
Domestic help†			
Increased	21 (18.4)	33 (26.6)	0.62 (0.34–1.16)
Same/decreased	93 (81.6)	99 (73.4)	
Care assistance‡			
Increased	65 (57.5)	75 (60)	0.90 (0.54–1.51)
Same/decreased	48 (42.5)	50 (40)	
Informal care∫			
Increased	41 (36.3)	37 (30.3)	1.31 (0.76–2.25)
Same/decreased	72 (63.7)	85 (69.7)	

* No Care Dependency Score was available for 3 patients.

† No data were available about domestic help for 8 patients.

‡ No data were available about care assistance for 8 patients.

∫ No data were available about informal care for 11 patients.

**Table 5:** Efficacy of intervention on quality of life.

Short Form-36 Admission-discharge scores per domain*	Intervention Group N = 117	Usual-Care Group N = 129	OR (95% CI)
**Physical Function, n (%)**			
Same/better	26 (22.8)	29 (23.2)	1.02 (0.56–1.87)
Worse	88 (77.2)	96 (76.8)	
**Social Function, n (%)**			
Same/better	51 (44.7)	57 (45.6)	1.04 (0.62–1.72)
Worse	63 (55.3)	68 (54.4)	
**Role Physical, n (%)**			
Same/better	41 (36.0)	48 (30.4)	1.11 (0.66–1.88)
Worse	73 (64.0)	77 (61.6)	
**Role Emotional, n (%)**			
Same/better	55 (48.2)	74 (59.2)	1.56 (0.93–2.60)
Worse	59 (51.8)	51 (40.8)	
**Mental Health, n (%)**			
Same/better	71 (62.3)	71 (56.8)	0.80 (0.47–1.34)
Worse	43 (37.7)	54 (43.2)	
**Vitality, n (%)**			
Same/better	43 (37.7)	49 (39.2)	1.07 (0.63–1.79)
Worse	71 (62.3)	76 (60.8)	
**Bodily Pain, n (%)**			
Same/better	57 (50)	41 (32.8)	0.49 (0.29–0.82)
Worse	57 (50)	84 (67.2)	
**General Health, n (%)**			
Same/better	67 (58.8)	68 (54.4)	0.84 (0.50–1.40)
Worse	47 (41.2)	57 (45.6)	
**Health Change, n (%)**			
Same/better	74 (64.9)	96 (72.0)	1.39 (0.80–2.41)
Worse	40 (35.1)	35 (28.0)	

* No Short Form-36 score was available for seven patients, while 14 patients died during hospital stay.

#### Incidence of delirium

In total, 260 patients were analysed for the primary outcome measure. Delirium was found to have occurred in 31 of these patients (11.9%). There was no significant difference between the incidence of delirium in the intervention group and in the usual-care group (9.4% vs. 14.3%, OR: 0.63, 95% CI: 0.29–1.35). The relative risk of delirium in the intervention group versus the usual-care group was 0.66. The severity of delirium as measured by the highest value of the DRS-R-98 did not differ significantly between the intervention group and the usual-care group (9 [5–30] vs. 15 [5–29], p = 0.11).

The delirium incidence rates varied per category of surgical procedure with 1.5% (1/65), 14.6% (7/48) and 15.6% (23/147) in the minor, intermediate and major groups respectively (see Table 1 for classification of interventions). The delirium incidence differed most between the groups of patients undergoing an intermediate intervention (21.4% in the control group and 5% in the intervention group, OR: 0.14, 95%CI: 0.02–1.75).

#### Postoperative complications

There was no significant difference between the groups in the number and type of complications that occurred (Table 6). Cardiovascular complications (31.5% in the intervention group and 27.8% in the control group) and pulmonary complications (24.4% in the intervention group and 20.3% in the control group) were the most common. Wound infection, electrolyte disturbance, urinary retention and ileus/gastroparesis also occurred frequently (around 10%).

**Table 6:** Number of patients with complications according to study group.

Postoperative complication	Intervention group N = 127	Control group N = 133	p-value (1-sided)
Pulmonary complication, n (%)	31 (24.4)	27 (20.3)	0.22
Neurological complication, n (%)	8 (6.3)	8 (6.0)	0.46
Cardiovascular complication, n (%)	40 (31.5)	37 (27.8)	0.26
Thromboembolic complication, n (%)	1 (0.8)	0 (0)	0.15
Bleeding, n (%)	11 (8.7)	6 (4.5)	0.09
Wound infection, n (%)	13 (10.2)	12 (9.0)	0.37
Wound dehiscence, n (%)	4 (3.1)	4 (3.0)	0.47
Urinary tract infection, n (%)	8 (6.3)	7 (5.3)	0.36
Anastomotic leakage, n (%)	5 (3.9)	2 (1.5)	0.11
Pressure ulcer, n (%)	5 (3.9)	7 (5.3)	0.31
Renal failure, n (%)	5 (3.9)	2 (1.5)	0.11
Electrolyte disturbance, n (%)	15 (11.8)	12 (9.0)	0.23
Fall, n (%)	4 (3.1)	2 (1.5)	0.19
Urinary retention, n (%)	15 (11.8)	12 (9.0)	0.23
Ileus/gastroparesis, n (%)	9 (7.1)	14 (10.5)	0.16

In the intervention group, 42 patients (33.1%) had more than one postoperative complication versus 38 patients (28.6%) in the control group (OR: 1.24, 95% CI: 0.73–2.10).

#### Mortality

Two patients died before the operation. Fourteen patients died during the hospital stay. There was no significant difference between the intervention group and the usual-care group (7.9% versus 3.0%, OR: 2.76, 95% CI: 0.84–9.03).

#### Length of hospital stay

The median length of the hospital stay was eight days in both groups, ranging from one to 135 days in the intervention group and from one to 44 days in the usual-care group. The percentage of patients who stayed in hospital longer than eight days did not differ between the groups (49.6% versus 42.9%, OR: 1.28 [0.77–2.12]). Of the 260 patients analysed for the primary outcome measure, 76 (29.2%) stayed in the intensive-care unit postoperatively, 39 (30.7%) in the intervention group and 37 (27.8%) in the usual-care group. Of these 76 patients, the median stay was one day for both groups, ranging from one to nine days in the intervention group and from one to 22 days in the usual-care group (p = 0.35).

#### Return to preoperative living situation and care

In the intervention group, 67.3% (76 out of 113) returned to an independent preoperative living situation on discharge versus 79.1% in the usual-care group (87 out of 110). This was a significant difference (OR: 1.84, 95% CI: 1.01–3.37).

#### Care dependency

On discharge most patients were more care dependent than before the operation. There was no significant difference between the groups (74.1% versus 75.6%, OR: 0.93, 95% CI: 0.52–1.65).

#### Quality of life

There was no significant difference between the groups in most aspects of the SF-36 scale, although patients in the intervention group did report significantly less bodily pain at discharge than at admission compared with the usual-care group (OR: 0.49, 95% CI: 0.29–0.82).

## Discussion

This randomized controlled trial could not provide evidence that a geriatric liaison intervention decreases postoperative delirium in frail elderly patients undergoing surgery for a solid tumour. Nor did the study find an effect of the intervention on the severity of delirium.

Furthermore, there was no significant difference between the groups in the number and type of complications, mortality, care dependency, length of hospital stay and length of ICU stay. The quality of life differed only in the area of bodily pain on the SF-36 in favour of the intervention group. More patients in the usual-care group returned to an independent preoperative living situation than in the intervention group.

Other non-pharmacological multicomponent intervention studies aimed at decreasing delirium in hospitalized elderly have shown varying results. Most studies have investigated the incidence of postoperative delirium in elderly hip-fracture patients, and some of these have found a significant reduction in delirium incidence [30], [31], severity [30], [32] and duration [32], while others have shown no effect on either delirium incidence or socioeconomic outcome parameters [31], [32]. The same applies to studies in geriatric and general medicine populations. The studies of Inouye (1999) and Caplan (2007) have both shown a significant reduction in delirium incidence; the effect of an intervention on the severity and duration of delirium remains controversial, however [29], [33]. The latter study indicated cost effectiveness, however, and showed a significant positive effect on ADL and MMSE scores even though no significant effect was shown on readmissions, discharge to residential care and length of hospital stay.

In summary, our negative results correspond with previous studies, and there are several possible reasons for our outcomes.

### Primary outcome measure

This study was aimed at improving postoperative outcomes in frail elderly cancer patients. Postoperative delirium was chosen as the primary outcome measure given its association with increased morbidity and mortality, persistent functional and cognitive decline, longer hospital stay, higher rates of nursing home placement and increased health care costs [13]–[17]. Moreover, delirium is a short-term outcome, reducing the likelihood of bias.

Most previous delirium prevention studies included orthopaedic patients (usually hip-fracture patients) or patients from an acute care unit. There is broad experience of different models of shared orthopaedic and geriatric care for elderly hip-fracture patients. The positive effect of a daily geriatric consultative service has been described, but there is a trend towards integrated care as the most effective model [34]. In such care, a geriatrician is added to the orthopaedic team to oversee the management of the patient from admission until discharge. A positive effect has been seen here on mortality, length of hospital stay and mean time to surgery. The effect on medical complication rates is not clear, however, because a wide range of definitions of complications is used in the included studies. The benefits of a consultative service on request and an orthopaedic consultative service on the geriatric ward are less clear. Up to now, evidence for any benefits of consultation-based management of delirium in any setting is lacking. This implies that the intervention model chosen in this study has failed, but that it may be effective when applied in an integrated care model.

The present study is unique in terms of the selected population. Delirium incidence rates in this study were unexpectedly low in both the intervention group and the usual-care group. In the population studied the relative incidence decreased by 34% (14.3% vs. 9.4%) with an overall incidence rate of 11.9%. Although this is an impressive overall reduction, the study was underpowered due to the low overall incidence of delirium. The power calculation was based on delirium incidence rates in orthopaedic, abdominal and cardiac surgery patients. To our knowledge, data on delirium incidence rates in the geriatric oncological surgical patients have not previously been reported.

There may be several explanations for this low incidence rate. First, it implies a high standard of care for frail elderly patients in the participating hospitals. Each hospital already had specialized geriatric care available before the start of this trial. Although standard consultation for frail elderly patients was not part of the routine treatment, there was already some awareness in the medical and nursing staff of the risks involved in treating frail elderly patients.

Patients with severe cognitive impairment were unable to comply with the study protocol and were excluded; however, this group is at the highest risk of the development of delirium. In addition, the study not only included patients undergoing major surgery, but also patients undergoing minor and intermediate surgical procedures. It is well known that surgical procedures for breast cancer and dermal tumours result in few and mostly local complications, even in patients over the age of 80 [35], [36]. For example, Ansaloni et al. found a delirium incidence rate of 1.6% for salpingovariectomy, quadrantectomy, mastectomy, axillary lymph node dissection and thyroidectomy versus 33.3% for gastric resection and gastrointestinal perforation closure [11]. The results of the present study show that this also applies to frail patients. A probable explanation for this difference is that a stress response in combination with elevated inflammatory markers provoked by surgery or infectious states plays an important role in the pathogenesis of postoperative delirium [37], [38]. One can imagine that this response is more distinct in patients undergoing major surgery. Another explanation might be that patient characteristics differed per tumour type with respect to, for example, sex, nutritional status and quality of life. These characteristics may have influenced the delirium risk.

In this study, patients were selected with the GFI, which was originally developed to screen for level of frailty [22]. Frail persons have decreased ability to compensate for disruptions in homeostasis due to a loss of reserves. Frailty is associated with an increased risk of falls, hospitalization, institutionalization, disability and death in community-dwelling older adults [39]–[41], as well as with an increased risk of post-operative complications (including delirium), length of hospitalization and inability to be discharged home in hospitalized patients [3]–[5]. The GFI distinguishes itself from most other frailty measurement instruments in that it includes not only physical but also cognitive, psychological and social items. Based on literature suggesting that frailty and delirium may be different clinical expression of a shared vulnerability to stress, we expected that patients considered frail by the GFI would be at higher risk of postoperative delirium [42]. Given the low delirium incidence rate in this study, the GFI was probably not an accurate selection method. For future delirium prevention studies, we would recommend to select patients at high risk of postoperative delirium based on earlier identified risk factors [6], [18], [43]–[45].

Finally, the nature of the geriatric intervention was broadly defined in a pre-operative and post-operative checklist. The geriatric checklist was recorded and adhered to per patient, but analysing these extensive data proved to be very complicated. For example, at the beginning of the study we tried to record drugs usage for all participants, but this proved to be unfeasible due to the voluminous data. In retrospect, we could have focused on deliriogenic drugs only. These are important limitations of the study and a focus for future multicomponent delirium prevention studies.

### Contamination

**Figure 2:**
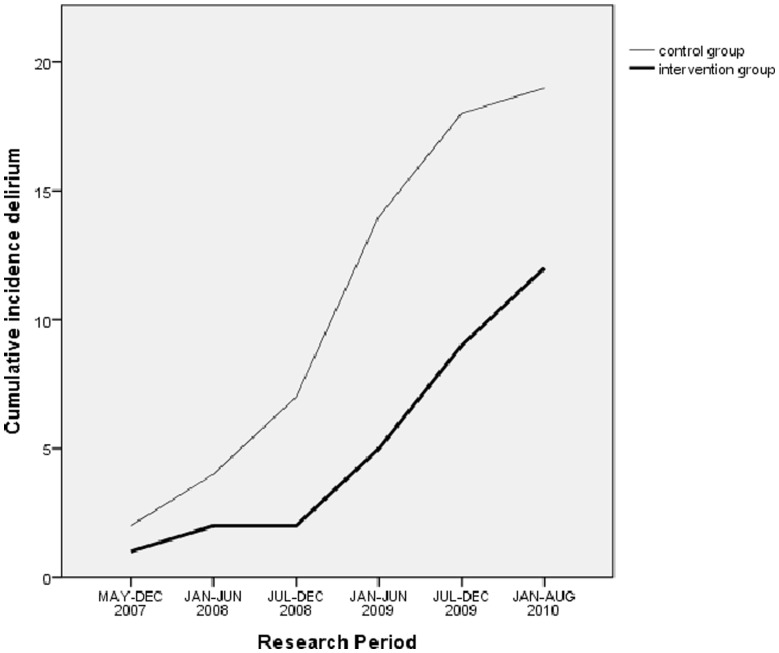
Cumulative delirium incidence in the control group and research period.

As mentioned before, the ward and research nurses were not blinded to the group to which a patient was randomized. This could lead to contamination, that is, additional interventions in the standard care group. In the case of contamination, one would expect a decrease in the difference in the incidence rate of delirium between the groups as the study progressed. As the lines in Figure 2 are not convergent, this argues against contamination.

### Secondary outcomes

There was no difference between the groups in terms of postoperative complications, mortality, care dependency post-discharge and length of hospital stay. More patients in the usual-care group returned to the preoperative living situation. Patients in the intervention group who lived independently preoperatively were more often (temporarily) discharged to a nursing home than such patients in the control group. A possible reason was that geriatric care may lead to rehabilitation in a nursing home after discharge.

The effect of the intervention on the quality of life was only seen in the domain of bodily pain of the SF-36. The clinical importance of this outcome is unclear.

### Selection and inclusion of frail elderly

In a separate paper, we presented an overview of problems we encountered while conducting this study [45]. The first problem is that the selection of patients is extremely important in this research population. Patients who are too frail or too fit should be excluded to optimize internal validity (the need to focus the study group to maximize the chances of detecting any impact of the intervention). However, eligibility criteria should not be too strict with respect to external validity (the ability to generalize to a larger population). For example, patients unable to understand questionnaires were excluded, although patients with decreased cognitive abilities are at high risk of developing delirium. Furthermore, patients undergoing surgery for a superficial tumour (skin, breast) were included in the study, although they are at low risk of developing postoperative delirium. Both criteria may have lowered the delirium incidence rate in our study and reduced the likelihood of showing the intervention to be effective.

However, the main problem was that the actual inclusion rate fell short of expectations. This was due to: 1) Limited physical and cognitive reserve of frail elderly patients, making participation and extra visits to the hospital a burden for them; 2) Difficulty in understanding written information and information given over the phone; and 3) Insufficient awareness of the study by health-care professionals. To increase inclusion rates, follow-up measurements were taken during a home visit. To overcome barriers to understanding written information and information given over the phone, patients were informed face to face and questionnaires were completed in an interview format. To increase awareness, posters, pencil and sweets with the logo of the study were distributed, and the study protocol was repeatedly explained to new staff. Moreover, checks were made as to whether possible eligible patients coming to the hospital were indeed screened for participation. These measures increased inclusion rates but also caused an increased time investment and consequently extra staffing costs.

Finally, the drop-out rate (12.5%) was higher than the expected 10%, which is a widely used drop-out rate in research with adults. This should be considered in future research in this population.

Further analyses are needed to determine the cost-effectiveness and long-term effect of the intervention on related postoperative outcomes such as mortality, quality of life, care dependency and living situation.

### Conclusion

Within this study, geriatric liaison intervention for the prevention of postoperative delirium in frail patients in a general oncological surgical population has not proven to be effective. Certain limitations to the study design, such as patient selection, may have played a role. Future intensive collaboration between surgeons and geriatricians may be warranted to improve postoperative outcomes in frail elderly cancer patients.

## Supporting Information

Checklist S1 **CONSORT Checklist.**
(DOC)

Protocol S1 **Trial Protocol.**
(PDF)

## References

[1] International Institute for Applied Systems Analysis (2002) Europe: population by age groups, 1950–2050. Available: www.iiasa.ac.at/research.

[2] Monson K, Litvak DA, Bold RJ (2003) Surgery in the aged population: surgical oncology. Arch Surg 138 10: 1061–1067. 14557121 10.1001/archsurg.138.10.1061

[3] Dasgupta M, Rolfson DB, Stolee P, Borrie MJ, Speechley M (2009) Frailty is associated with postoperative complications in older adults with medical problems. Arch Gerontol Geriatr 48: 78–83. 18068828 10.1016/j.archger.2007.10.007

[4] Audisio RA, Pope D, Ramesh HS, Gennari R, van Leeuwen BL, et al (2008) Shall we operate? Preoperative assessment in elderly cancer patients (PACE) can help. A SIOG surgical task force prospective study. Crit Rev Oncol Hematol 65: 156–63. 18082416 10.1016/j.critrevonc.2007.11.001

[5] Leung JM, Tsai TL, Sands LP (2011) Brief report: Preoperative frailty in older surgical patients is associated with early postoperative delirium. Anesth Analg 112: 1199–1201. 21372278 10.1213/ANE.0b013e31820c7c06 PMC3081949

[6] Dasgupta M, Dumbrell AC (2006) Preoperative risk assessment for delirium after noncardiac surgery: a systematic review. J Am Geriatr Soc 54: 1578–89. 17038078 10.1111/j.1532-5415.2006.00893.x

[7] Robinson TN, Raeburn CD, Tran ZV, Angles EM, Brenner LA, et al (2009) Postoperative delirium in the elderly: risk factors and outcomes. Ann Surg 249: 173–8. 19106695 10.1097/SLA.0b013e31818e4776

[8] Brouquet A, Cudennec T, Benoist S, Moulias S, Beauchet A, et al (2010) Impaired mobility, ASA status and administration of tramadol are risk factors for postoperative delirium in patients aged 75 years or more after major abdominal surgery. Ann Surg 251: 759–65. 20224380 10.1097/SLA.0b013e3181c1cfc9

[9] Koebrugge B, Koek HL, van Wensen RJ, Dautzenberg PL, Bosscha K (2009) Delirium after abdominal surgery at a surgical ward with a high standard of delirium care: incidence, risk factors and outcomes. Dig Surg 26: 63–8. 19169032 10.1159/000194947

[10] Tei M, Ikeda M, Haraguchi N, Takemasa I, Mizushima T, et al (2010) Risk factors for postoperative delirium in elderly patients with colorectal cancer. Surg Endosc 23: 2135–9. 10.1007/s00464-010-0911-7 20177939

[11] Ansaloni L, Catena F, Chattat R, Fortuna D, Franceschi C, et al (2010) Risk factors and incidence of postoperative delirium in elderly patients after elective and emergency surgery. Br J Surg 97: 273–80. 20069607 10.1002/bjs.6843

[12] van der Mast RC, Roest FH (1996) Delirium after cardiac surgery: a critical review. J Psychosom Res 41: 13–30. 8887815 10.1016/0022-3999(96)00005-0

[13] Leslie DL, Marcantonio ER, Zhang Y, Leo-Summers L, Inouye SK (2008) One-year health care costs associated with delirium in the elderly population. Arch Intern Med 168: 27–32. 18195192 10.1001/archinternmed.2007.4 PMC4559525

[14] McCusker J, Cole M, Dendukuri N, Belzile E, Primeau F (2001) Delirium in older medical inpatients and subsequent cognitive and functional status: a prospective study. CMAJ 165: 575–83. 11563209 PMC81415

[15] Inouye SK, Rushing JT, Foreman MD, Palmer RM, Pompei P (1998) Does delirium contribute to poor hospital outcomes? A three-site epidemiologic study. J Gen Intern Med 13: 234–42. 9565386 10.1046/j.1525-1497.1998.00073.x PMC1496947

[16] O'Keeffe S, Lavan J (1997) The prognostic significance of delirium in older hospital patients. J Am Geriatr Soc 45: 174–8. 9033515 10.1111/j.1532-5415.1997.tb04503.x

[17] Witlox J, Eurelings LS, de Jonghe JF, Kalisvaart KJ, Eikelenboom P, et al (2010) Delirium in elderly patients and the risk of postdischarge mortality, institutionalization, and dementia: a meta-analysis. JAMA 304: 443–51. 20664045 10.1001/jama.2010.1013

[18] Marcantonio ER, Goldman L, Mangione CM, Ludwig LE, Muraca B, et al (1994) A clinical prediction rule for delirium after elective noncardiac surgery. JAMA 271: 134–9. 8264068

[19] Hempenius L, van Leeuwen BL, van Asselt DZB, Hoekstra HJ, Wiggers T, et al (2010) Structured analyses of interventions to prevent delirium. Int J Ger Psych 26: 441–450. 10.1002/gps.2560 20848577

[20] Al-Aama T, Brymer C, Gutmanis I, Woolmore-Goodwin SM, Esbaugh J, et al (2011) Melatonine decreases delirium in elderly patients: a randomized, placebo-controlled trial. Int J Ger Psych 26: 687–94. 10.1002/gps.2582 20845391

[21] Schuurmans H, Steverink N, Lindenberg S, Frieswijk N, Slaets JP (2004) Old or frail: what tells us more? J Gerontol A Biol Sci Med Sci 59: M962–M965. 15472162 10.1093/gerona/59.9.m962

[22] Steverink N, Slaets JPJ, Schuurmans H, van Lis M (2009) Measuring frailty: development and testing of de Groningen Frailty Indicator (GFI). Gerontologist 41: 236–7.

[23] Kalisvaart KJ (2005). Primary prevention of delirium in the elderly. Amsterdam: University of Amsterdam, PhD thesis.

[24] McHorney CA, Ware JE Jr, Lu JF, Sherbourne CD (1994) The MOS 36-item Short-Form Health Survey (SF-36): III. Tests of data quality, scaling assumptions, and reliability across diverse patient groups. Med Care 32: 40–66. 8277801 10.1097/00005650-199401000-00004

[25] Dijkstra A, Buist G, Dassen T (1996) Nursing-care dependency. Development of an assessment scale for demented and mentally handicapped patients. Scand J Caring Sci 10: 137–43. 9060783 10.1111/j.1471-6712.1996.tb00326.x

[26] Tombaugh TN, McIntyre NJ (1992) The mini-mental state examination: a comprehensive review. J Am Geriatr Soc 40: 922–35. 1512391 10.1111/j.1532-5415.1992.tb01992.x

[27] Schuurmans MJ, Shortridge-Baggett LM, Duursma SA (2003) The Delirium Observation Screening Scale: a screening instrument for delirium. Res Theory Nurs Pract 17: 31–50. 12751884 10.1891/rtnp.17.1.31.53169

[28] Trzepacz PT, Mittal D, Torres R, Kanary K, Norton J, et al (2001) Validation of the Delirium Rating Scale-revised-98: comparison with the delirium rating scale and the cognitive test for delirium. J Neuropsychiatry Clin Neurosci 13: 229–242. 11449030 10.1176/jnp.13.2.229

[29] Inouye SK, Bogardus ST Jr, Charpentier PA, Leo-Summers L, Acampora, et al (1999) A multicomponent intervention to prevent delirium in hospitalized older patients. N Engl J Med 340: 669–76. 10053175 10.1056/NEJM199903043400901

[30] Marcantonio ER, Flacker JM, Wright RJ, Resnick NM (2001) Reducing delirium after hip fracture: a randomized trial. J Am Geriatr Soc 49: 516–22. 11380742 10.1046/j.1532-5415.2001.49108.x

[31] Wong T, Niam D, Bruce JJ, Bruce DG (2005) Quality project to prevent delirium after hip fracture. Australas J Ageing 24: 174–177.

[32] Millisen K, Foreman MD, Abraham IL, De Geest S, Godderis J, et al (2001) A nures-led interdisciplinary intervention program for delirium in elderly hip-fracture patients. J Am Geriatr Soc 49: 523–532. 11380743 10.1046/j.1532-5415.2001.49109.x

[33] Caplan GA, Harper EL (2007) Recruitment of volunteers to improve vitality in the elderly: the REVIVE study. Intern Med J 37: 95–100. 17229251 10.1111/j.1445-5994.2007.01265.x

[34] Kammerlander C, Roth T, Friedman SM, Suhm N, Luger TJ, et al (2010) Ortho-geriatric service - a literature review comparing different models. Osteoporos Int 21: S637–S646. 21058004 10.1007/s00198-010-1396-x

[35] Paradela S, Pita-Fernandez S, Pena C, Fernandez-Jorge B, Garcia-Silva J, et al (2010) Complications of ambulatory major dermatological surgery in patients older than 85 years. J Eur Acad Dermatol Venereol 24: 1207–13. 20337810 10.1111/j.1468-3083.2010.03628.x

[36] Rao VS, Jameel JK, Mahapatra TK, McManus PL, Fox JN, et al (2007) Surgery is associated with lower morbidity and longer survival in elderly breast cancer patients over 80. Breast J 13: 368–73. 17593041 10.1111/j.1524-4741.2007.00444.x

[37] Maclullich AM, Ferguson KJ, Miller T, de Rooij SE, Cunningham C (2008) Unravelling the pathophysiology of delirium: a focus on the role of aberrant stress responses. J Psychosom Res 65: 229–38. 18707945 10.1016/j.jpsychores.2008.05.019 PMC4311661

[38] van Munster BC, Bisschop PH, Zwinderman AH, Korevaar JC, Endert E, et al (2010) Cortisol, interleukins and S100B in delirium in the elderly. Brain Cogn 74: 18–23. 20580479 10.1016/j.bandc.2010.05.010

[39] Fried LP, Tangen CM, Walston J, Newman AB, Hirsch C, et al (2001) Frailty in older adults: evidence for a phenotype. J Gerontol A Biol Sci Med Sci 56 3: M146–56. 11253156 10.1093/gerona/56.3.m146

[40] Morley JE, Perry HM 3rd, Miller DK (2002) Editorial: Something about frailty. J Gerontol A Biol Sci Med Sci 57 11: M698–704. 12403796 10.1093/gerona/57.11.m698

[41] Rockwood K, Mitnitski A (2007) Frailty in relation to the accumulation of deficits. J Gerontol A Biol Sci Med Sci 62 7: 722–7. 17634318 10.1093/gerona/62.7.722

[42] Quinlan N, Marcantonio ER, Inouye SK, Gill TM, Kamholz B, et al (2011) Vulnerability: the crossroads of frailty and delirium. J Am Geriatr Soc 59 2: S262–8. 22091571 10.1111/j.1532-5415.2011.03674.x PMC3233987

[43] Elie M, Cole MG, Primeau FJ, Bellavance F (1998) Delirium risk factors in elderly hospitalized patients. J Gen Intern Med 13 3: 204–12. 9541379 10.1046/j.1525-1497.1998.00047.x PMC1496920

[44] Inouye SK, Charpentier PA (1996) Percipitating factors for delirium in hospitalized elderly persons. Predictive model and interrelationship with baseline vulnerability. JAMA 275 11: 852–7. 8596223

[45] Hempenius L, Slaets JPJ, Boelens AM, Van Asselt DZB, De Bock GH, et al (2013) Inclusion of frail elderly in clinical trials: Solutions to the problems. J Geriatr Oncol 4 1: 26–31. 24071489 10.1016/j.jgo.2012.08.004

