# Supplementary material for: Outcomes of a Geriatric Liaison Intervention to Prevent the Development of Postoperative Delirium in Frail Elderly Cancer Patients: Report on a Multicentre, Randomized, Controlled Trial
Source: PLoS One. 2013 Jun 19;8(6):e64834. doi: 10.1371/journal.pone.0064834 (PMC3686791; doi:10.1371/journal.pone.0064834)
Supplement: Protocol S1 — Trial Protocol. (PDF) [file pone.0064834.s002.pdf]

Categorie A

Hanzeplein 1 Postbus 30 001, 9700 RB Groningen

Universitair Medisch Centrum Groningen

Chirurgie
Hoofd Prof. dr. T. Wiggers

Aan
mr. J.W.P. de Vroedt,
ambtelijke secretaris
Medisch Etische Toetsingscommissie
De Brug 7.069
UMCG

Telefoon (050) 36 12301
Fax (050) 36 11745

Bijlage(n) diversen
Kenmerk CHI06.1092/TW/ajh

Datum 26 oktober 2006
Onderwerp indiening protocol

Geachte collega,

Hierbij bied ik u het protocol "A randomized controlled trial of geriatric liaison intervention in frail surgical oncology patients" ter beoordeling aan.

Het betreft hier een ZonMw goedgekeurde studie. Gaarne zouden wij eerst beoordeling in het UMCG krijgen waarna het in tweede instantie ter beoordeling kan komen van de participerende centra.

Ik wil u verzoeken ontheffing van de verzekering te verlenen aangezien het onderzoek door zijn aard zonder risico's voor de deelnemende patiënten moet zijn.

Bijgevoegd zijn de noodzakelijke documenten.

Met vriendelijke groet,

T. Wiggers

umcg

Categorie B

Sluiten

Formulier voor medisch-ethische beoordeling en registratie

ABR-formulier, versie februari 2006

Onderzoeksdossiernummer

ABR Nummer	**15136**	Status	**Definitief**
Versie	**01**	Status per	**25-10-2006**
Jaar	**06**		
Dossiernummer	**NL15136.042.06**		

A. Sectie - Openbaar maken gegevens medisch wetenschappelijk onderzoek

A1. De CCMO streeft naar meer transparantie over (de beoordeling van) medischwetenschappelijk onderzoek met mensen, geslachtscellen, embryo's en foetussen in utero. De verrichter (opdrachtgever/sponsor) kan hier aangeven toestemming te verlenen voor de openbaarmaking van de antwoorden op de vragen gemarkeerd met een wereldbol en de samenvatting bij dit formulier via de CCMO-website, dan wel de openbaarmaking te weigeren:

- (●) de verrichter verleent de CCMO toestemming voor de bovengenoemde openbaarmaking
- ( ) de verrichter verleent de CCMO géén toestemming voor de bovengenoemde openbaarmaking

B. Sectie - Administratief

B1. Betreft het onderzoek met geneesmiddelen (inclusief gentherapie, somatische celtherapie, GGO's, zie verdere toelichting) als bedoeld in de Wet medisch-wetenschappelijk onderzoek met mensen (WMO)?

- ( ) ja
- (●) nee

B2. Houdt het onderzoek verband met een eerder door een erkende METC of door de CCMO beoordeelde studie of is het onderzoek reeds eerder bij een erkende METC ter beoordeling voorgelegd?

- ( ) ja, het onderzoek houdt verband met - of is het vervolg op - een eerder beoordeelde studie
- ( ) ja, het onderzoek is eerder ter beoordeling aan een erkende METC of de CCMO voorgelegd (stuur kopie besluit mee)
- (●) nee

B3. Is het protocol reeds door een andere instantie op zijn wetenschappelijke aspecten beoordeeld?

- ( ) ja (stuur kopie van beoordeling mee)
- (●) nee

B4. Is het protocol (nog) in een ander openbaar trial register geregistreerd?

- ( ) ja
- (●) nee

B5. Naam indiener/contactpersoon voor de oordelende toetsingscommissie

Achternaam indiener/contactpersoon	Wiggers
Titel en voorletters	prof dr T
Tussenvoegsel	
Type Organisatie/Bedrijf	Universitair Medisch Centrum
Organisatie/Bedrijf	Universitair Medisch Centrum Groningen

Afdeling	
Intern adres	
Adres	PO BOX 30001
Postcode en plaats	9700RB Groningen
Land	Nederland
Telefoon	050-3612301
Fax	050-3614873
E-mail	t.wiggers@chir.umcg.nl

B6. Is de indiener werkzaam bij de opdrachtgever/sponsor (verrichter) van het onderzoek?

(•) Ja ( ) Nee

C. Sectie - Onderzoek

C1. Volledige titel van het onderzoek

C1a. In het Engels

A randomized controlled trial of geriatric liaison intervention in frail surgical oncology patients

C1b. In het Nederlands

Een prospectief gerandomiseerde studie naar het effect van een geriatrische interventie bij kwetsbare kankerpatienten die een chirurgische behandeling nodig hebben.

C2. Verkorte titel van het onderzoek/acroniem

C2a. In het Engels

geriatric intervention

C2b. In het Nederlands

geriatrische interventie

C3. Trefwoorden (maximaal 4, plaats elk trefwoord op een aparte regel)

C3a. In het Engels

cancer
frail patients
surgical oncology
geriatric

C3b. In het Nederlands

kanker
kwestbare patienten
chirurgische oncologie
geriatrisch

C4. Beschrijf het belang van het onderzoek en de beoogde toepassing van de resultaten (verwijs eventueel naar de relevante pagina's in het protocol).

pagina 9 introduction en rationale

C5. Wat is de hoofdvraagstelling van het onderzoek?

kan het delirium bij kwetsbare oudere patienten die geopereerd worden voor kanker voorkomen worden door een geriatrische interventie

C6. Betreft het onderzoek een multicenter-onderzoek?

( ) nee
(•) ja - alleen in Nederland
( ) ja - internationaal binnen de Europese Unie
( ) ja - internationaal ook buiten de Europese Unie

C7. Is er bij multicenter-onderzoek sprake van een coördinerend onderzoeker?

* (x) ja namelijk — Naam onderzoeker: Barbara van Leeuwen
( ) nee

C8. Wie is/zijn medisch verantwoordelijk voor de proefpersonen die deelnemen aan het onderzoek

prof dr T Wiggers
dr R Vree
dr JPEN Pierie

C9. In welk centrum/welke centra (incl. huisartsenpraktijken) in Nederland wordt het onderzoek uitgevoerd? Geef per centrum op: aantal proefpersonen, naam hoofdonderzoeker, naam onafhankelijk arts.

Centrum Informatie

Centrum	Proefpersonen	Hoofdonderzoeker	Onafhankelijk arts
Diaconessenhuis Leiden	98	dr R Vree	dr J Plukker
Medisch Centrum Leeuwarden	98	dr JPEN Pierie	dr J Plukker
Universitair Medisch Centrum Groningen	98	prof dr T Wiggers	dr J Plukker

C10. Betreft het onderzoek met:

- [x] mensen
- [ ] geslachtscellen
- [ ] (rest-)embryo's
- [ ] foetussen in utero

C11. Beoogd totaal aantal proefpersonen/(rest)embryo's/foetussen in utero:

C11a. In Nederland
294

C12. Op welke pagina van het onderzoeksprotocol wordt een onderbouwing gegeven van het aantal proefpersonen/(rest-)embryo's/foetussen in utero?

pag 13

C13. Onderzoeksgebied

- [ ] etiologie
- [ ] organisatorisch/zorgonderzoek
- [ ] diagnostiek
- [ ] preventie
- [x] therapie
- [ ] veiligheid
- [ ] werkzaamheid
- [ ] farmacokinetiek
- [ ] farmacodynamiek
- [ ] bio-equivalentie
- [ ] dosis-respons
- [ ] farmacogenomics
- [ ] farmaco-economie
- [ ] anders

C14. Type onderzoek

( ) observationeel onderzoek zonder invasieve metingen

- ( ) observationeel onderzoek met invasieve metingen
- (•) interventie-onderzoek

C19. Is er sprake van (nog) een andere interventie dan met geneesmiddelen zoals bedoeld in de Wet Medisch Wetenschappelijk Onderzoek met Mensen (zie toelichting)?

- ( ) Ja
- (•) Nee

C20. Is/zijn er (een) controlegroep (en)?

- (•) ja
- ( ) nee
- [ ] ja, geneesmiddel
- [ ] ja, placebo
- [x] ja, geen inteventie
- [ ] ja, anders

C21. Betreft het een gerandomiseerd onderzoek?

- (•) ja
- ( ) nee

namelijk

- [x] open
- [ ] enkelblind
- [ ] dubbelblind
- [ ] parallel
- [ ] cross-over
- [ ] anders

C22. Op welke klasse(n) van aandoeningen heeft het onderzoek betrekking (maximaal 3)

- [ ] hartaandoeningen
- [ ] congenitale, familiaire en genetische aandoeningen
- [ ] bloed- en lymfestelsel aandoeningen
- [ ] zenuwstelsel aandoeningen
- [ ] oogaandoeningen
- [ ] evenwichtsorgaan- en ooraandoeningen
- [ ] ademhalingsstelsel-, thorax- en mediastinumaandoeningen
- [ ] maagdarmstelselaandoeningen
- [ ] nier- en urinewegaandoeningen
- [ ] huid- en onderhuidaandoeningen
- [ ] skeletspierstelsel- en bindweefselaandoeningen
- [ ] endocriene aandoeningen
- [ ] voedingsstoornissen en metabole ziekten
- [ ] infecties en parasitaire aandoeningen
- [ ] letsels, intoxicaties en verrichtingscomplicaties
- [x] neoplasmata, benigne, maligne en niet-gespecificeerd (incl cysten en poliepen)
  diverse enplaats niet-gespecificeerde neoplasmata maligne en niet-gespeci
- [x] chirurgische en medische verrichtingen
  therapeutische verrichtingen en ondersteunende behandeling NEG
- [ ] bloedvataandoeningen
- [ ] algemene aandoeningen en aandoeningen op de plek van toediening
- [ ] zwangerschap, perinatale periode en puerperium

☐ sociale omstandigheden
☐ immuunsysteemaandoeningen
☐ lever- en galaandoeningen
☐ voortplantingsstelsel- en borstaandoeningen
☐ psychische stoornissen
☐ overig, namelijk

C23.Geef twee synoniemen voor de aandoening die bestudeerd wordt, waarvan tenminste één lekenterm.

In het Engels:	cancer in the elderly
In het Nederlands:	kanker bij de oudere

C24. Beoogde start- en einddatum van het onderzoek

C24a. Start Datum (dd-mm-jjjj)	01-01-2007
C24b. Eind Datum (dd-mm-jjjj)	30-09-2009

D. Sectie - Proefpersonen

D1. Is er een proefpersonenverzekering conform de WMO-eisen afgesloten of wordt aan de oordelende toetsingscommissie ontheffing gevraagd?

○ proefpersonenverzekering is afgesloten bij verzekeringsmaatschappij
◉ ontheffing van de verzekering wordt gevraagd
○ niet van toepassing - onderzoek valt onder de Embryowet en niet onder de WMO

D2. Gezonde proefpersonen en/of patiënten

☐ Gezonde proefpersonen
☑ Patiënten

Aantal 298

D3. Welke specifieke populaties worden voor deelname aan het onderzoek benaderd?

oudere patienten die na afnemen van een vragenlijst kwetsbaar zijn

D4. Voornaamste inclusiecriteria

D4a. In het Engels
frail elderly patients in need for a cancer operation
D4b. In het Nederlands
kwetsbare oudere patienten die voor een kankerbehandeling geopereerd worden

D5. Voornaamste exclusiecriteria

D5a. In het Engels
unable to comply outcome questionaires
no compiance for follow-up
D5b. In het Nederlands
onvermogen vragenlijsten te beantwoorden
miet in staat om vervolgd te worden na de interventie

D6. Bij welke categorie proefpersonen wordt het onderzoek uitgevoerd (meerdere antwoorden mogelijk)

☑ 18 jaar of ouder en wilsbekwaam **(ga naar vraag D10)**
☐ 18 jaar of ouder en wilsonbekwaam **(ga naar vraag D7)**
☐ 12 t/m 17 jaar en in staat tot het geven van geïnformeerde toestemming **(ga naar vraag D8)**
☐ 12 t/m 17 jaar en niet in staat tot het geven van geïnformeerde toestemming (wilsonbekwaam) **(ga naar vraag D7)**

☐ jonger dan 12 jaar **(ga naar vraag D8)**

D10. Verkeren (sommige) proefpersonen in een afhankelijkheidssituatie ten opzichte van de onderzoeker of degene die de deelnemers werft? (lees de toelichting voor voorbeelden wanneer er sprake kan zijn van een afhankelijkheidssituatie)

○ ja
◉ nee

D11. Waaruit bestaat de vergoeding voor de proefpersonen?

☑ geen vergoeding
☐ reiskosten
☐ financiële vergoeding (in Euro's)
☐ andere vergoeding

D12. Is deze vergoeding afhankelijk van bepaalde voorwaarden, bijvoorbeeld het voltooien van (een deel van) het onderzoek?

○ ja (motiveer)
◉ nee
○ niet van toepassing

E. Sectie - Voor- en nadelen

E1. Wordt er bij dit onderzoek een rechtstreeks therapeutisch effect beoogd bij de proefpersonen / patiënten?

◉ ja (therapeutisch onderzoek)
○ nee (niet-therapeutisch onderzoek)

E1a. Zo ja, geef kort aan waaruit dit therapeutisch effect bestaat.
minder delirium

E2. Waaruit bestaat de belasting van het onderzoek (en een eventueel daaraan voorafgaande keuring) voor de proefpersonen?

Tijdbeslag	per bezoek	30 minuten
	totaal	6
	totale duur van de studie voor de individuele proefpersoon	180

E3. Worden de proefpersonen in verband met het onderzoek in het ziekenhuis opgenomen of wordt een opname verlengd?

○ ja - het verblijf het ziekenhuis/instituut wordt in verband met het onderzoek verlengd
○ ja - ze worden voor het onderzoek in het ziekenhuis/instituut opgenomen
◉ nee

E4. Beschrijf in hoeverre proefpersonen worden onderworpen aan handelingen dan wel een gedragswijze krijgen opgelegd, zoals vragenlijst, interviews, lichamelijk/psychologisch onderzoek, ontzegging, dieet (voor invasieve ingrepen: zie vraag E6)

vragenlijsten

E5. Worden de proefpersonen getest op bepaalde aandoeningen/condities?

◉ ja (motiveer)
○ nee

namelijk kwetsbaarheid via de frailty index

E6. Welke extra (invasieve) ingrepen (anders dan bij de standaard behandeling) moeten de proefpersonen in het kader van het onderzoek ondergaan:

☑ Niet van toepassing

E7. Welke maatregelen zijn genomen om (eventuele) risico's van de bijkomende ingrepen te vermijden en nadelige gevolgen te beperken?

er zijn geen bijkomende ingrepen

E8. Hoe is de spreiding van de ingrepen in de tijd, met name afname van bloed en ander lichaamsmateriaal (verwijs eventueel naar de flow chart in het protocol)?

zie blz 20

E9. Geef aan welke risico's er voor proefpersonen zijn verbonden aan deelname aan het onderzoek.

geen

E9a. Geef op grond van uw eigen afweging aan waarom het uitvoeren van het onderzoek, in het licht van de belasting en/of risico's die voor proefpersonen aan deelname verbonden zijn, gerechtvaardigd is?

deze studie moet gaan aantonen dat een geintegreerde geriatrische benadering bij kwetsbare oudere patienten standaard ingevoerd kan worden in Nederland. in feite gebeurt de voorgestelde interventie al incidenteel

E10. Indien het onderzoek bij minderjarige en/of wilsonbekwame proefpersonen wordt uitgevoerd en geen direct therapeutisch effect wordt beoogd: waarom kunnen belasting en risico's als minimaal worden beschouwd? (Verwijs eventueel naar de relevante pagina's in het protocol.)

☑ niet van toepassing

E11. Kan de eventuele therapie na beëindiging van het onderzoek worden voortgezet?

○ ja (motiveer)

○ nee (motiveer)

◉ niet van toepassing

E12. Heeft deelname aan het onderzoek voor de proefpersoon tot gevolg dat van de standaardbehandeling of -diagnostiek kan worden afgeweken of deze kan worden uitgesteld?

○ ja

◉ nee

○ niet van toepassing

F. Sectie - Informatie en privacy

F1. Hoe worden de proefpersonen geworven en door wie (onderzoeker, behandelend arts, andere persoon) wordt de proefpersoon/wettelijke vertegenwoordiger geïnformeerd en om toestemming gevraagd?

door de behandeled arts. deze geeft mondelinge en schriftelijke informatie

F2. Hoeveel bedenktijd krijgen de proefpersonen/wettelijke vertegenwoordigers om te beslissen over deelname?

1-2 dagen

F3. Wordt de huisarts, behandelend specialist en/of apotheker van de proefpersoon geïnformeerd over diens deelname aan het onderzoek?

◉ ja (de proefpersoon dient hiervoor toestemming te geven)

○ nee

F4. Worden persoonsgegevens gecodeerd?

○ ja

◉ nee

omdat het neit nodig is om terug te gaan naar de individuele patientgegevens

F5. Hoe wordt het lichaamsmateriaal gedurende het onderzoek bewaard?

- ( ) in tot de proefpersoon herleidbare vorm
- ( ) in niet tot de proefpersoon herleidbare vorm (volledig geanonimiseerd)
- (•) niet van toepassing

F6. Wordt afgenomen lichaamsmateriaal na afloop van het onderzoek vernietigd?

- ( ) ja
- ( ) nee (motiveer)
- (•) niet van toepassing

F7. Kunnen proefpersonen na afloop van het onderzoek opnieuw benaderd worden (bijvoorbeeld voor nader onderzoek of follow-up)?

- ( ) ja
- (•) nee

G. Sectie - Financieel

G1. Door welke geldstroom wordt het onderzoek gefinancierd?

- [ ] eerste geldstroom (Geld van Ministerie van OC&W aan universiteiten)
- [ ] tweede geldstroom (NWO of KNAW), namelijk:
- [x] derde geldstroom (anders dan 1e of 2e geldstroom, zoals collectebusfondsen, Europese Unie, vakministeries of bedrijven), namelijk:

ZonMW

G2. Wordt het onderzoek (mede) gefinancierd door de industrie/bedrijven?

- ( ) ja - door de industrie/bedrijf zoals is opgegeven bij vraag B6 (opdrachtgever van het onderzoek)
- ( ) ja - (ook) door andere industrie/bedrijven dan de opdrachtgever
- (•) nee

G3. Ontvangt de arts/onderzoeker persoonlijk (afgezien van zijn/haar normale salaris) een vergoeding voor de uitvoering van het onderhavige onderzoek?

- ( ) ja
- (•) nee

G4. Heeft/hebben de onderzoeker(s) gedurende de afgelopen vijf jaar op een of andere wijze een persoonlijke financiële relatie (gehad) met de verrichter/sponsor van het huidige onderzoek?

- ( ) ja (licht toe)
- (•) nee

H. Sectie - Publicatiebeleid

H1. Zijn door de opdrachtgever/subsidiegever bepaalde voorwaarden gesteld aan het openbaar maken van de onderzoeksresultaten?

- ( ) ja (licht toe)
- (•) nee

I. Sectie - Indiening en beoordeling

I1. Selecteer de toetsingscommissie die het oordeel geeft in de zin van de WMO

Commissie
METC Universitair Medisch Centrum Groningen (Groningen)

J. Sectie - Aanvullende opmerkingen

Aanvullende opmerkingen

K. Sectie - Samenvatting

Titel onderzoek

Een prospectief gerandomiseerde studie naar het effect van een geriatrische interventie bij kwetsbare kankerpatienten die een chirurgische behandeling nodig hebben.

Achtergrond van het onderzoek:

Kanker is een ziekte van de oudere. De operatieve behandeling gaat gepaard met een verhoogde morbiditeit en zelfs mortaliteit. Deze verhoogde kans op complicaties komt vooral voor bij de kwetsbare oudere patient Preventie van het voorkomen van het delier is het gevolg van een interventie van een geriatrisch team

Doel van het onderzoek:

Het verminderen van de incidentie van het postoperatief delier bij kwetsbare ouderen opgenomen voor

oncologische chirurgie. De interventie bestaat uit een medebehandeling door een team gespecialiseerd in de ouderengeneeskunde en gericht op delierpreventie en een optimaal postoperatief functioneel herstel.

Onderzoeksopzet:

Het betreft een prospectief gerandomiseerde studie

Onderzoekspopulatie:

In deze studieopzet worden patiënten ouder dan 65 jaar, die een operatie ondergaan vanwege een solide maligne tumor, perioperatief door een specialistisch team voor ouderengeneeskunde medebehandeld. Met behulp van de Groninger Frailty Indicator zullen patiënten met een score groter dan 3 worden geselecteerd. De verwachting is dat deze kwetsbare ouderen een hoog risico op een postoperatief delier hebben en tevens baat hebben bij de medebehandeling.

Interventie (indien van toepassing):

In nauwe samenwerking met de chirurg en anesthesioloog zal het geriatrische team een geprotocoleerde bijdrage leveren aan het behandelplan tijdens de opname gericht op delierpreventie en optimaal functioneel herstel. De geriater zal de supervisor zijn van het geriatrisch team.

Primaire onderzoeksvariabelen/uitkomstmaten:

De primaire uitkomstmaat is het optreden van een postoperatief delier. Dit wordt bepaald met behulp van de Delirium Observation Screening Scale (DOS) en de criteriumdiagnose van de DSM-IV.
We verwachten een afname in de incidentie van het postoperatieve delier in deze hoog-risico groep. Door een vermindering van de incidentie en/of de ernst van het delier verwachten wij in de interventiegroep een positief effect op de afhankelijkheid van zorg bij ontslag, gemeten met de Care Dependency Scale. Wij verwachten ook een toename van belangrijke determinanten van kwaliteit van leven gemeten met de Physical en Mental Component Summary measure van de SF-36 op het moment van ontslag en na 3 maanden follow-up.

Secundaire onderzoeksvariabelen/uitkomstmaten (indien van toepassing):
zie boven

Omschrijving en inschatting van belasting en risico (indien van toepassing):

De meer intensieve zorg kan nar onze mening niet leiden tot risico's voor de patienten

K2.Engelse Samenvatting

Title of the study
A randomized controlled trial of geriatric liaison intervention in frail surgical oncology patients

Background of the study:
Rationale: It has been shown that a multicomponent intervention can decrease the occurrence of delirium in older patients (Inouye et al 1999). Geriatric liaison teams are well trained in implementing best-supportive care programs for elderly. In standard care geriatricians are not involved in pre-operative screening of patients and perioperative care and will only be consulted after major complications (delirium) or functional losses have occurred.

Objective of the study:
Objective: The primary objective of this study is to show that early detection of geriatric patients at risk of preventable functional decline following a surgical procedure under general anesthesia for a solid tumor, combined with a geriatric liaison intervention will decrease the occurrence of delirium and consequent morbidity and mortality, without an increase in costs.

Study design:
Study design: This is a multicenter prospective randomized clinical trial.

Study population:
Study population: Patients over 65 years of age admitted to the Department of Surgery of the participating centres for the surgical or combined cancer treatment (surgery / radiation /chemotherapy / hormonal therapy) of a solid tumor will be included in this study

Intervention (if applicable):
Intervention: The intervention entails participation of a geriatric nurse and geriatrician in the perioperative treatment of the oncogeriatric surgical patient.

Primary study parameters/outcome of the study:
Main study parameters/endpoints: The main endpoint is the cumulative incidence of delirium (measured with the Delirium Observation Scale and the DSM IV criteria) up to 10 days postoperatively. Secondary endpoints are: returning to the pre-operative living situation within 3 months postoperatively,the Physical Component Summary measure (PCS) of the SF-36, the Mental Component Summary measure (MCS) of the SF-36, complications during hospital stay including mortality, care Dependence Scale at discharge. Direct health care and non-health care costs will be used as economic indicators

Secundary study parameters/outcome of the study (if applicable):
see above

Nature and extent of the burden and risks associated with participation, benefit and group relatedness (if applicable):
The participating patients will have to complete 2 questionnaires at inclusion in the study which will take about 30 minutes in total. During their hospital stay they will be asked to complete several questionnaires which will take 15 minutes daily on average. Also 3 months postoperatively they will be asked to complete a questionnaire which takes 15-30 minutes on average. The Hb value will be taken from the routine blood samples and no extra blood samples or diagnostic tests will be performed on the participating patients unless their medical condition requires this (e.g. to rule out dehydration). Although changes in diet or medication may be made in the study group these are not expected to cause an extra burden or discomfort to the participating patients. No experimental drugs will be used during this study.

ONDERTEKENING

De verrichter en indiener verklaren hierbij:
a. het formulier (en samenvatting) volledig en naar waarheid te hebben ingevuld;
b. de antwoorden op de vragen uit het ABR-formulier niet in strijd zijn met het bijbehorende onderzoeksdossier en onderzoekscontract

Naar waarheid getekend, door de verrichter (=opdrachtgever)

datum 26/10/06

Handtekening
naam T. Wiggers
functie hoofd afdeling chirurgie

door de indiener

datum 26/10/06

Handtekening
naam T. Wiggers
functie chirurg

Sluiten

Aanbiedingsformulier METc UMCG voor beoordeling onderzoek als bedoeld in de Wet medisch-wetenschappelijk onderzoek met mensen (WMO)

Ondergetekende is in het Universitair Medisch Centrum Groningen **verantwoordelijk onderzoeker** voor het onderzoek met als titel:

A randomized controlled trial of geriatric liaison intervention in frail surgical oncology patients [volledige titel onderzoek invullen]

Verklaart ten behoeve van de beoordeling als bedoeld in de Wet medisch-wetenschappelijk onderzoek met mensen (WMO) door de METc UMCG het volgende:

Ten behoeve van de beoordeling worden de volgende **documenten** ingediend.

omschrijving document	***versiennr.***	***versiedatum***	***cat****
ABR-formulier, dossiernummer 15136	01	25-10-2006	**B**
Aanbiedingsbrief		26-102006	**B**
Protocol (inclusief informatie en vragenlijsten)		25-10-2006	**C**
CV		25-10-2006	**H**
Toestemmingsbrief ZonMw			**K**
Lijst deelnemende centra met hoofdonderzoeker		25-10-06	**I**
			....
			....
			....
			....
			....
			....
			....
			....
			....
			....
			....
			....
			....
			....
			....
			....
			....
			....
			....
			....
			....
			....

* = categorie (A, B, C, D, E, F, G, H, I, J of K) als bedoeld in de notitie 'standaard onderzoeksdossier' van de Centrale Commissie Mensgebonden Onderzoek

☐ Bovenstaand is niet voldoende ruimte voor vermelding van alle documenten. Overige documenten staan vermeld op bijlage bij dit formulier (onder vermelding van omschrijving van het document, versienummer en –datum en categorie)

- Het betreft een onderzoek met (één of meer) **geneesmiddelen**
  ☒ nee
  ☐ ja
  Zo ja, heeft afstemming plaatsgevonden met de **Ziekenhuisapotheek** van het UMCG?
  ☐ nee
  ☐ ja, met [naam contactpersoon invullen]
  (contactpersonen: mevrouw drs. M. Maurer, Ziekenhuisapotheek UMCG, tel. 14127 of mevrouw drs. B.H.W. Molmans, Ziekenhuisapotheek UMCG, tel. 10376)

- Het betreft een onderzoek met één of meer **medische hulpmiddelen**
  ☒ nee
  ☐ ja
  Zo ja, is er toestemming van de **Terzake Deskundige** als bedoeld in de Wet op de medisch hulpmiddelen om het (de) medische hulpmiddel(len) in het UMCG te gebruiken
  (contactpersoon: A. Nederhoed, Dienst AV&M, tel. 13836)
  ☐ ja
  ☐ nee omdat

- Het betreft een onderzoek waarbij **radioactieve straling** wordt toegepast
  ☒ nee
  ☐ ja
  Zo ja, is de stralenbelasting voor proefpersonen berekend door een **stralingsdeskundige**
  ☐ ja, door [naam stralingsdeskundige invullen]
  ☐ nee, omdat

- De **verwerking van de persoonsgegevens** ten behoeve van het onderzoek is gemeld bij de **Functionaris voor de Gegevensbescherming van het UMCG.**
  (contactpersoon: mevrouw dr. G.J. Meijer, Directoraat Zorgfaciliteiten, tel. 19833)
  ☒ ja
  ☐ nee, omdat

- Bij de uitvoering van het zijn de volgende **organisatieonderdelen** van het UMCG betrokken en geïnformeerd.[betrokken organisatieonderdelen kiezen en/of invullen]

Medische afdelingen

anesthesiologie	naam contactpersoon	prof dr L Aarts
chirurgie	naam contactpersoon	prof dt T Wiggers
chirurgie	naam contactpersoon	prof dr HJ Hoekstra
geriatrie	naam contactpersoon	prof dr J Slaets

Zorgfaciliteiten

....	naam contactpersoon	
....	naam contactpersoon	
	naam contactpersoon	

Anders

Epidemiologie	naam contactpersoon	dr T de Bock
	naam contactpersoon	
	naam contactpersoon	

- De **betrokken organisatieonderdelen** zijn bereid en in staat te zijn hun **medewerking aan het onderzoek te verlenen**
  ☐ nee
  ☒ ja

Is (zijn) een **lid (leden) van de METc UMCG** betrokken bij het opstellen van het onderzoeksprotocol of de uitvoering van het onderzoek?

- [x] nee
- [ ] ja

Zo ja, welk(e) lid (leden) van de METc UMCG?

[naam invullen]
[naam invullen]

- **Nadere opmerkingen**

De coordinerende onderzoeker is mevrouw van Leeuwen. Zij verblijft in verband met een KWF fellowship deze maanden in Zweden. Hoofdonderzoekers zijn collega Slaets en ondergetekende. Samen hebben wij de subsidie bij ZonMw verkeregen.

Ondertekening:

Naam **verantwoordelijk onderzoeker**	De coordinerende onderzoeker is T. Wiggers
Organisatieonderdeel	chirurgie
Telefoonnummer (intern)	12301
E-mailadres	t.wiggers@chir.umcg.nl
Datum	25-10-2006

Handtekening verantwoordelijk onderzoeker:

Naam **hoofd van organisatieonderdeel** waar verantwoordelijk onderzoeker werkzaam is	T. Wiggers

Verklaart dat

- de verantwoordelijk onderzoeker deskundig en bekwaam is voor het uitvoeren van het onderzoek;
- het organisatieonderdeel berekend is op een gedegen uitvoering van het onderzoek;
- alle personen die participeren in de uitvoering van het onderzoek zullen worden voorgelicht over de inhoud van het onderzoeksprotocol;
- het organisatieonderdeel berekend is op het uitvoeren van het onderzoek;
- andere onderzoeken zodanig afgestemd worden dat de uitvoering van deze onderzoeken en dit onderzoek niet in gevaar komt;
- het onderzoek overigens op een verantwoorde en zorgvuldige wijze zal worden uitgevoerd.

Datum 26-10-2006

Handtekening hoofd organisatieonderdeel:

NB: In geval van zg. industrieel onderzoek zal bij de opdrachtgever een tarief in rekening worden gebracht voor de beoordeling van het onderzoek door de METc UMCG.

Categorie C

A randomized controlled trial of geriatric liaison intervention in frail surgical oncology patients

A randomized controlled trial of geriatric liaison intervention in frail surgical oncology patients

Protocol ID	Geriatric intervention
Short title	
Version	
Date	October 2006
Coordinating investigator/project leader	*Barbara van Leeuwen*<br>***blvleeuwen@hetnet.nl***<br>*050-5891968*
Principal investigator(s) (in Dutch: hoofdonderzoeker/uitvoerder)	*Prof T. Wiggers. Afdeling Heelkunde, Universitair Medisch Centrum Groningen, Postbus 4195, 9700 ED Groningen*<br>*Prof J Slaets, Geriatrie Universitair Medisch Centrum Groningen*<br>*Prof HJ Hoekstra, Chirurgie, Universitair Medisch Centrum Groningen*<br>*Dr T de Bock, Epidemiologie , Universitair Medisch Centrum Groningen*
Multicenter research: per site	*Dr. R. Vree, Afdeling Heelkunde, Diaconessenhuis, Houtlaan 55, 2334 CK Leiden*<br>*Dr. J.P.E.N. Pierie, Afdeling Heelkunde, Medisch Centrum Leeuwarden, Henri Dunantweg 2, postbus 888, 8901BR Leeuwarden*
Sponsor (in Dutch: verrichter/opdrachtgever)	*ZonMw, Nederlandse organisatie voor gezondheidsonderzoek en zorginnovatie*<br>*Postbus 93245, 2509 AE Den Haag, tel: 070-3495111, fax: 070-3495100, info@zonmw.nl >*

Independent physician(s)	*Dr.JT.M. Plukker , Afdeling Heelkunde, Universitair Medisch Centrum Groningen*

PROTOCOL SIGNATURE SHEET

Name	Signature	Date
For non-commercial research,<br>Head of Department:<br>Prof. Dr. T. Wiggers		
Coordinating Investigator/Project leader/Principal Investigator:<br>Dr. B.L. van Leeuwen		
Dr. J.P.E.N. Pierie		
Dr. R. Vree		

TABLE OF CONTENTS

1. OBJECTIVES 14
2. STUDY DESIGN 15
   i. Inclusion phase 1: 3 months 15
   ii. Inclusion phase 2: 3-33 months 15
   iii. Follow up and analysis phase 3: 33-36 months 15
3. STUDY POPULATION 16
   3.1 Population (base) 16
   3.2 Inclusion criteria 16
   3.3 Exclusion criteria 16
   3.4 Sample size calculation 16
4. TREATMENT OF SUBJECTS 18
   4.1 Investigational treatment 18
5. METHODS 24
   5.1 Study parameters/endpoints 24
      5.1.1 Main study parameter/endpoint 24
      5.1.2 Secondary study parameters/endpoints 24
   5.2 Randomisation, blinding and treatment allocation 24
   5.3 Study procedures 24
   During treatment 25
   After the end of treatment (Follow-up): 26
   3 months postoperatively the following data will be recorded 26
   5.4 Withdrawal of individual subjects 28
   5.5 Replacement of individual subjects after withdrawal 28
   5.6 Follow-up of subjects withdrawn from treatment 28
6. SAFETY REPORTING 29
   6.1 Section 10 WMO event 29
   6.2 Adverse and serious adverse events 29
   6.3 Follow-up of adverse events 30
   6.4 Data Safety Monitoring Board (DSMB) 30
7. STATISTICAL ANALYSIS 31
   7.1 Descriptive statistics 31
8. ETHICAL CONSIDERATIONS 32
   8.1 Regulation statement 32
   8.2 Recruitment and consent 32
   8.3 Benefits and risks assessment, group relatedness 33
   8.4 Compensation for injury 33
9. ADMINISTRATIVE ASPECTS AND PUBLICATION 33
   9.1 Handling and storage of data and documents 33
   9.2 Amendments 34
   9.3 Annual progress report 34
   9.4 End of study report 34

9.5 Public disclosure and publication policy 35
10. REFERENCES 36

Verwi

Verwi

LIST OF ABBREVIATIONS AND RELEVANT DEFINITIONS

ABR	ABR form (General Assessment and Registration form) is the application form that is required for submission to the accredited Ethics Committee (ABR = Algemene Beoordeling en Registratie)
AE	Adverse Event
AR	Adverse Reaction
CA	Competent Authority
CCMO	Central Committee on Research Involving Human Subjects
CV	Curriculum Vitae
DSMB	Data Safety Monitoring Board
EU	European Union
EudraCT	European drug regulatory affairs Clinical Trials GCP Good Clinical Practice
IB	Investigator's Brochure
IC	Informed Consent
IMP	Investigational Medicinal Product
IMPD	Investigational Medicinal Product Dossier
METC	Medical research ethics committee (MREC); in Dutch: medisch ethische toetsing commissie (METC)
(S)AE	Serious Adverse Event
SPC	Summary of Product Characteristics (in Dutch: officiële productinfomatie IB1-tekst)
Sponsor	The sponsor is the party that commissions the organisation or performance of the research, for example a pharmaceutical<br>company, academic hospital, scientific organisation or investigator. A party that provides funding for a study but does not commission it is not regarded as the sponsor, but referred to as a subsidising party.
SUSAR	Suspected Unexpected Serious Adverse Reaction
Wbp	Personal Data Protection Act (in Dutch: Wet Bescherming Persoonsgevens)
WMO	Medical Research Involving Human Subjects Act (Wet Medisch-wetenschappelijk Onderzoek met Mensen

SUMMARY

Rationale: It has been shown that a multicomponent intervention can decrease the occurrence of delirium in older patients (Inouye et al 1999). Geriatric liaison teams are well trained in implementing best-supportive care programs for elderly. In standard care geriatricians are not involved in pre-operative screening of patients and perioperative care and will only be consulted after major complications (delirium) or functional losses have occurred.

Objective: The primary objective of this study is to show that early detection of geriatric patients at risk of preventable functional decline following a surgical procedure under general anesthesia for a solid tumor, combined with a geriatric liaison intervention will decrease the occurrence of delirium and consequent morbidity and mortality, without an increase in costs.

Study design: This is a multicenter prospective randomized clinical trial.

Study population: Patients over 65 years of age admitted to the Department of Surgery of the participating centres for the surgical or combined cancer treatment (surgery / radiation /chemotherapy / hormonal therapy) of a solid tumor will be included in this study

Intervention: The intervention entails participation of a geriatric nurse and geriatrician in the perioperative treatment of the oncogeriatric surgical patient.

Main study parameters/endpoints: The main endpoint is the cumulative incidence of delirium (measured with the Delirium Observation Scale and the DSM IV criteria) up to 10 days postoperatively. Secondary endpoints are: returning to the pre-operative living situation within 3 months postoperatively,the Physical Component Summary measure (PCS) of the SF-36, the Mental Component Summary measure (MCS) of the SF-36, complications during hospital stay including mortality, care Dependence Scale at discharge. Direct health care and non-health care costs will be used as economic indicators

Nature and extent of the burden and risks associated with participation, benefit and group relatedness: The participating patients will have to complete 2 questionnaires at inclusion in the study which will take about 30 minutes in total. During their hospital stay they will be asked to complete several questionnaires which will take 15 minutes daily on average. Also 3 months postoperatively they will be asked to complete a questionnaire which takes 15-30 minutes on average. The Hb value will be taken from the routine blood samples and no extra blood samples or diagnostic tests will be performed on the participating patients unless their medical condition requires this (e.g. to rule out dehydration). Although changes in diet or medication may be made in the study group these are not expected to cause an extra burden or discomfort to the participating patients. No experimental drugs will be used during this study.

INTRODUCTION AND RATIONALE

Cancer is a disease of the elderly: 50% of all new cases of cancer and 60% of all cancer deaths occur in patients over 65 years. It is expected that the prevalence of cancer in the Dutch population will increase with 35% (breast cancer) to 60% (lung cancer) in the next 20 years with an increase in the absolute number of patients older than 65 years. The estimate number of cancer patients older than 65 in The Netherlands is 91.000. For 2020 the prediction will be 137.000 patients. About 57% of the patients with a solid tumor are treated with surgery, mostly as part of a combined modality treatment including (neo) adjuvant radiation and/or adjuvant chemotherapy and/or hormonal treatment. Although postoperative mortality is acceptable in selected elderly patients (Damhuis 2005), fewer chances of proper diagnosis and good quality of care are currently offered to the older cancer patients (Repetto, 2003; Vercelli et al 2000; Audisio et al 2004; Fentiman et al, 1996). Hospitalization of older patients is often associated with the development of disability that is potentially preventable. It is estimated that approximately 35% of patients aged 75 and older develop a new disability after hospitalization or suffer functional declines. This holds true for the Netherlands (Olde Rikkert, 2004), as well as for other countries (e.g., Rothschild et al., 2000; Sager et al., 1996). Institutionalization of these patients leads to further costs, not to mention loss of quality of life. Moreover, it is estimated that a considerable part of these adverse effects in hospitalized patients are preventable. Estimates range from 35% (e.g., Olde Rikkert, 2004) to 65% (e.g., Brennan et al., 1991). It is paramount to early identify the most complex cases at risk of developing new functional losses in an attempt to prevent their occurrence during the cancer treatment and the course of hospitalization. (Wymenga et al, 2001). After an uneventful recovery the prognosis of cancer patients is similar to the recovery in young patients. More patients will be able to receive proper adjuvant treatment. As 50% to 70% of elderly patients in hospital do not have an increased risk for adverse outcomes due to geriatric problems, geriatric interventions should be targeted not to elderly per se but to frail elderly only. At the University Medical Center Groningen a case-finding instrument based on these principles has been developed. The Groningen Frailty Indicator (GFI) (Schuurmans et al., 2004; Steverink et al., 2001; Slaets, 2004) has been applied in a wide range of patients and elderly populations. We did a prospective study on 85 consecutive admissions for oncological surgery in the UMCG. 30% of these patients had a GFI greater than 3 (mean score 3, median score 2). About one third of the patients over 65 years old in this study will have a GFI score greater than 3. They are considered as frail elderly. The effects of intervention will be studied only in this subgroup of patients in comparison to standard care.

The importance of delirium.

Delirium is a common psychiatric disorder in hospitalized elderly. It is a serious and potentially preventable medical condition. It is an acute confusional state characterized by an alteration of consciousness with reduced ability to focus, sustain or shift attention (Francis 1990). Reported incidence in literature varies widely from less than 10 up to 50% of hospitalized patients and it is often misdiagnosed. The occurrence of postoperative delirium in elderly patients is associated with an increase in morbidity and mortality (Van Hemert 1994). Delirium is also a costly disorder, increasing length of hospital stay and healthcare

costs by up to 25% (Franco 2001). Based on literature it seems fair to assume a 30% incidence of postoperative delirium in frail elderly cancer patient postoperatively. There are two steps in the process of diagnosing delirium. The first step is to screen every patient at risk by nurses on a day tot day basis. Nurses play a crucial role in the recognition of delirium as they have frequent contacts with the patients and are in a good position to observe behavioural changes. We will use the Delirium Observation Scale (DOS) as a screening instrument for delirium. This observation rated screening scale was designed for the early recognition of delirium based on observations by the nurses during regular care. The DOS has a high internal consistency with a Cronbach's alpha 0,92 to 0,96 (M. Schuurmans et.al). The sensitivity of the algorithm based on 13 DOS-items against the clinical DSM-IV diagnosis was 100% with a specitivity of 68%. (Schuurmans). The negative predictive value of the DOS algorithm in this study was also 100%. The nurses in the study will be trained to use the DOS. A score higher than 3 indicates delirium. The second step is to make the formal diagnosis of delirium by a medical specialist (geriatrician or psychiatrist) on the base of the DSM-IV criteria. The severity of delirium will be rated by the Delirium Rating Scale (DRS-R-98)

Motivation for the geriatric intervention

It has been shown that a multicomponent intervention can decrease the occurrence of delirium in older patients (Inouye et al 1999). Geriatric liaison teams are well trained in implementing best-supportive care programs for elderly. In standard care geriatricians are not involved in pre-operative screening of patients and perioperative care and will only be consulted after major complications (delirium) or functional losses have occurred. Combining the disease-oriented approach of surgeons with the patient-oriented approach of geriatricians we expect to decrease the occurrence of postoperative delirium in the frail elderly cancer patient treated for a solid tumor from 30% to 15%.

1. OBJECTIVES

The primary objective of this study is to show that early detection of geriatric patients at risk of preventable functional decline following a surgical procedure under general anesthesia for a solid tumor, combined with a geriatric liaison intervention will decrease the occurrence of delirium and consequent morbidity and mortality, without an increase in costs.

2. STUDY DESIGN

This is a multicenter prospective randomized clinical trial.

Patients eligible for the trial will be randomized to receive either perioperative intervention by the geriatric team or standard care.

Stratification

Randomization will be stratified by:

- tumor type e.g. a distinction will be made between tumors located in the chest (lung) or abdomen (liver, gastro-intestinal and pancreas) vs other tumors (breast, sarcoma, melanoma, thyroid).

Trial phases

i. Inclusion phase 1: 3 months

During the first 3 months all patients above 65 years will be assessed with the Groningen Frailty Index. For the patients with an index greater than 3 the geriatric liaison teams will be organized and the necessary steps to make the work of the teams comparable will take place. The geriatric liaison will prevent or detect adverse events in an earlier phase than usual.

ii. Inclusion phase 2: 3-33 months

In this period the randomization of the patients with a high level of frailty will take place. There will be no inclusions in this period for patients with a low level of frailty. We will randomize 132 patients in the care as standard group and 132 patients in the intervention group.

iii. Follow up and analysis phase 3: 33-36 months

Since the primary outcome is delirium, the analysis can start immediately after inclusion of the last patient. However, during the last period the follow up assessments will be completed in order to score the secondary endpoints. The final analysis and scientific output of the primary and secondary outcomes of the study will be completed.

3. STUDY POPULATION

3.1 Population (base)

Patients over 65 years of age admitted to the Department of Surgery of the participating centres for the surgical or combined cancer treatment (surgery / radiation /chemotherapy / hormonal therapy) of a solid tumor will be included in this study.

3.2 Inclusion criteria

- a score greater than 3 on the GFI.
- surgery is scheduled more than 24 hours after inclusion in the study as we feel this is the time necessary for the geriatric team to plan their perioperative measures
- surgery under general anesthesia.
- written informed consent given according to local regulations.

Patients can only be randomized in this trial once.

3.3 Exclusion criteria

- any psychological, familial, sociological or geographical condition potentially hampering compliance with the study protocol and follow-up schedule.
- Patient unable to comply with the outcome questionnaires

3.4 Sample size calculation

Sample size and stopping rules were computed using EAST- software.
The incidence of delirium in our population is assumed 30%. We expect to find an absolute reduction of 15 %. Group sample sizes of 2x132 to achieve 80% power to detect a difference of 0.15 with a significance level of 0.05 (one sided). With a drop out rate of 10% 294 patients are needed. There are at least 120 patients aged 65 and over treated for solid tumors in the surgical ward per center per year. If we assume that at least one third of these are frail elderly this results in 10 eligible frail patients every month. With an inclusion rate of around 90% we should be able to include 108 patients per year and the duration of accrual will be 30 months. This entails an inclusion rate of 3-4 patients per center per month.

4. TREATMENT OF SUBJECTS

4.1 Investigational treatment

The intervention entails participation of a geriatric nurse and geriatrician in the perioperative treatment of the oncogeriatric surgical patient. In the preoperative period the team will plan measures to prevent adverse effects. These may include dietary measures, changes in medication and medication to prevent delirium. In the postoperative period this will include close daily monitoring of the frail patients and when necessary adaptation of their treatment and care plans. Multi-component interventions to achieve best-supportive care in individual treatment plans will be implemented. These will be focused on electrolyte- and fluid levels, pain management, pharmacological clearance, miction and defecation, nutrition, early mobilization and rehabilitation, sleep, vision, hearing and cognition. Any change in treatment will occur in collaboration with the physician responsible for daily care on the surgical ward. The geriatrician will be the supervisor of the geriatric team. The Delirium observation scale (DOS) will be used to screen for delirium by the nurse (Schuurmans 2003) and the Delirium Rating Scale (DRS) will be used to measure the severity of the delirium (Trzepazc 1998, 2001). To ensure uniformity of geriatric intervention in participating centres a daily checklist will be used (adapted from Kalisvaart 2005). Every item on this checklist will have to be addressed and checked by the member of the geriatric team involved with the patient at that time.

Checklist geriatrisch team

Punten van aandacht:

- Oriëntatie
- Mobiliteit
- Vijf zintuigen
- Pijn
- Slaap
- Intake
- Familie
- Opvolgend advies

☐ = aankruisen indien verricht/aanwezig/van toepassing
D= dagelijks controleren/verrichten
A= punt van aandacht

Oriëntatie

☐ Bekende objecten van thuis aanwezig
☐ Bed aan raamzijde /hoek
D: kort gesprek, indien noodzakelijk jezelf voorstellen, geef informatie
A: is de kamer te druk voor deze patiënt (1-2 personen per kamer)

Cognitieve problemen

☐ Kalender
☐ Klok met zichtbare nummers of eigen klok
☐ Nachtlampje aan, indien niet mogelijk vraag de familie naar een nachtlampje
D: gesprek mbt oriëntatie (wie,wat,waarom,waar)
A: verpleeegkundige vertelt wie ze is, waarom ze er is en wat ze doet

Mobiliteit

D:verwijder CAD/infuus/drain indien mogelijk
D: loopt de patiënt rond?
☐ Ja geen actie ☐ Nee ☐ Fysiotherapie ingeschakeld

Zit de patiënt
☐ op stoel, gezicht naar raam of gang
☐ op stoel, buiten eigen kamer

Kan de patiënt het toilet op tijd bereiken?
☐ is het urinaal/postoel binnen handbereik?
☐ bel binnen handbereik (weet de patiënt hoe deze te gebruiken)

Zintuigen

Zien

Heeft patiënt een bril?

☐ Ja ☐ Nee

Is de bril van patiënt aanwezig?

☐ leesbril

☐ andere bril

Indien slecht zicht ondanks correctie

☐ gesproken boeken

☐ drank niet in glazen maar drinkbeker

A:observeer, benader de patiënt van de beste zijde

Horen

Heeft de patiënt moeite met het verstaan van gesproken woorden?

☐ Nee (geen actie)

☐ Ja ☐ gehoorapparaat aanwezig en in

A: Gebruik apparaat gedurende de dag
Begin het gesprek
Kijk de patiënt aan tijdens gesprek
Spreek rustig
Benader patiënt van beste zijde

Pijn

Heeft de patiënt pijn?

☐ Nee (geen actie)

☐ Ja

Pijnmedicatie gestart ☐ Ja ☐ Nee

A : Observeer de pijn, ernst en wanneer deze optreedt
Schakel een arts in indien noodzakelijk
Besteed aandacht aan de bijweringen van de analgetica

Slaap

Slaapt de patiënt goed?

☐ Ja (geen actie)

☐ Nee

A: Indien mogelijk meer activiteiten aanbieden gedurende de dag
Sederende medicatie? Vraag arts deze te veranderen indien noodzakelijk
Slaap medicatie. Vraag arts deze te stoppen of veranderen

Haal patiënt overdag uit bed
Activiteitenprogramma
Max 1 uur middagslaap
Indien sprake van angst/probleem mbt slaap-> ritueel voor het slapen gaan
Indien kamer te levendig evt andere kamer
Slaapmedicatie noodzakelijk?

Intake

Vloeibaar

Patiënt drinkt alles wat hij/zij krijgt

☐ Ja (geen actie) ☐ Nee (Stimuleer en bepaal ureum/creatinine na 2 dagen om hydratie te beoordelen)

Vast voedsel

Was de patiënt de afgelopen 5 dagen thuis een slechte eter?

☐ Ja (schakel diëtiste in) ☐ Nee (geen actie)

Smaakt het eten goed?

☐ Ja (geen actie) ☐ Nee (extra stimulatie)

D: defaecatie patroon, indien na 2 dagen geen defaecatie informeer arts

Eet alles

☐ Ja (geen actie) ☐ Nee (schakel diëtiste in, overweeg tussendoortjes)

Familie

Indien pt verward kan de verpleegkundige de familie vragen
Vaker op bezoek te komen
Krant mee te nemen en evt voor te lezen
Praat over het hier en nu, thuis

Medicatielijst

Voorgeschreven medicatie gecontroleerd ☐ Ja ☐ Nee

Veranderingen aangebracht ☐ Ja ☐ Nee

Veranderingen + reden:

Lab

Lab uitslagen bekeken ☐ Ja ☐ Nee ☐ n.v.t.

Extra lab aangevraagd ☐ Ja ☐ Nee

Reden:

Advies

Wordt advies van het team opgevolgd

☐ Ja (geen actie)

☐ Nee Goede reden-> geen actie

Twijfelachtig ->overleg met verpleegkundig team

5. METHODS

5.1 Study parameters/endpoints

5.1.1 Main study parameter/endpoint

The cumulative incidence of delirium (measured with the Delirium Observation Scale and the DSM IV criteria) up to 10 days postoperatively.

5.1.2 Secondary study parameters/endpoints

- Returning to the pre-operative living situation within 3 months postoperatively
- The Physical Component Summary measure (PCS) of the SF-36
- The Mental Component Summary measure (MCS) of the SF-36
- Complications during hospital stay including mortality
- Care Dependence Scale at discharge
- Direct health care and non-health care costs will be used as economic indicators.

5.2 Randomisation, blinding and treatment allocation

Minimisation will be used to randomize the patients.

Patients will be balanced per centre

Patients will be stratified according to:

- tumor location in chest (lung)or abdomen (liver, gastro-intestinal and pancreas) vs other tumors (breast, sarcoma, melanoma, thyroid)

5.3 Study procedures

In the outpatient clinic or a minimum of 24 hours prior to surgery the Groningen Frail Index Questionnaire will be applied to all patients over 65 years of age eligible for inclusion into the study. A research nurse will perform this task after the patient has been asked for informed consent. When a frailty index of >3 is found patients will be included in the study.
After obtaining informed consent the patient data will be filled in on the data form. These will include tumor type, surgery planned, age and sex. Then baseline values for the quality of life endpoints are obtained eg the Physical Component and Mental Component Summary Measure of the SF-36 and the Care Dependency scale. All these data are collected by a nurse. Preoperative Hb will be determined in the routine blood sample and no extra venapuncture is necessary.

During treatment

Daily screening for delirium in all patients up to discharge or 10 days postoperative with the delirium observation scale. On the first postoperative day the SF-36 score will also be measured. Patients in the study group may receive changes in their diet or medication as the geriatric intervention team sees fit but no experimental drugs or investigational procedures will be used. The changes made in the diet and medication of the patients depend on their medical situation and although the points of attention are standardized, the exact type of medication given or the dose can not be foreseen per individual patient.
The patient with a positive screen on the DOS in the intervention group will be seen by geriatricians or consultant psychiatrists who are not participating in the geriatric liaison-intervention team in order to apply the DSM-IV criteria for the diagnosis of delirium.(American Psychiatric Association).

During hospital stay:
Daily recording of:

- Diagnostic procedures (including X-ray, CT-scan)
- Antibiotics prescribed
- Drugs prescribed
- Consultation by medical specialist
- Complications:
    - Wound infection
    - Anastomotic leakage (when applicable)
    - respiratory complications (pneumonia, combined clinical and radiological diagnosis)
    - cardio vascular complications events including arrythmia and cardiac failure
    - neurological events (CVA, RIND)
    - renal failure (as defined by a need for hemodialysis)
    - urinary infection
    - Thrombembolic events
    - pressure sores
    - death.

Total duration of hospital stay will be recorded as will days spent in the ICU when applicable

At discharge:

The Physical Component Summary measure (PCS) of the SF-36
The Mental Component Summary measure (MCS) of the SF-36
Care Dependency Scale

After the end of treatment (Follow-up):

3 months postoperatively the following data will be recorded

Return to pre-operative living situation
The Physical Component Summary measure (PCS) of the SF-36
The Mental Component Summary measure (MCS) of the SF-36

DATA COLLECTION SCHEME STANDARD CARE GROUP

	At inclusion (outpatietnt or ward)	At admission	First post-operative day	Daily postoperatively	At Discharge	3 months postoperatively
Groningen Frailty Index	X					
Patient history (including smoking, drinking)	X					
Living situation	X					X
Medication used	X	X	X	X	X	X
SF-36		X	X		X	X
DOS		X	X	X		
packed cells given in 24 hours			X	X		
Curative or palliative intent of operation?			X			
Lab results (electrolytes, Hb, creatinin, leucocytes)	X		X	X		
Diagnostic procedures			X	X		
Days spent in the ICU			X	X		
CDS	X				X	
Comlpications			X	X		
Consultation by specialist			X	X		

DATA COLLECTION SCHEME INTERVENTION GROUP

	At inclusion (outpatietnt or ward)	At admission	First post-operative day	Daily postoperatively	At Discharge	3 months postoperatively
Groningen Frailty Index	X					
Patient history (including smoking, drinking)	X					
Living situation	X					X
Medication used	X	X	X	X	X	X
Geriatric checklist		X	X	X		
SF-36		X	X		X	X
DOS		X	X	X		
packed cells given in 24 hours			X	X		
Curative or palliative intent of operation?			X			
Lab results (electrolytes, Hb, creatinin, leucocytes)	X		X	X		
Diagnostic procedures			X	X		
Days spent in the ICU			X	X		
CDS	X				X	
Complications			X	X		
Consultation by specialist			X	X		

See appendix for exact content of tests

5.4 Withdrawal of individual subjects

Subjects can leave the study at any time for any reason if they wish to do so without any consequences. The investigator can decide to withdraw a subject from the study for urgent medical reasons.

5.5 Replacement of individual subjects after withdrawal

Individual subjects will not be replaced after withdrawal.

5.6 Follow-up of subjects withdrawn from treatment

Subjects withdrawn from treatment will receive standard care. Although we will not subject them to any tests, certain data such as length of hospital stay, complications and living situation 3 months postoperative will be recorded.

6. SAFETY REPORTING

6.1 Section 10 WMO event

In accordance to section 10, subsection 1, of the WMO, the investigator will inform the subjects and the reviewing accredited METC if anything occurs, on the basis of which it appears that the disadvantages of participation may be significantly greater than was foreseen in the research proposal. The study will be suspended pending further review by the accredited METC, except insofar as suspension would jeopardise the subjects' health. The investigator will take care that all subjects are kept informed.

6.2 Adverse and serious adverse events

Adverse events are defined as any undesirable experience occurring to a subject during a clinical trial, whether or not considered related to the investigational drug. All adverse events reported spontaneously by the subject or observed by the investigator or his staff will be recorded. No adverse effects of geriatric intervention are known to us.

A serious adverse event is any untoward medical occurrence or effect that at any dose results in death;

- is life threatening (at the time of the event);
- requires hospitalisation or prolongation of existing inpatients' hospitalisation;
- results in persistent or significant disability or incapacity;
- is a congenital anomaly or birth defect;
- is a new event of the trial likely to affect the safety of the subjects, such as an unexpected outcome of an adverse reaction, lack of efficacy of an IMP used for the treatment of a life threatening disease, major safety finding from a newly completed animal study, etc.

All SAEs will be reported to the accredited METC that approved the protocol, according to the requirements of that METC.

6.3 Follow-up of adverse events

All adverse events will be followed until they have abated, or until a stable situation has been reached. Depending on the event, follow up may require additional tests or medical procedures as indicated, and/or referral to the general physician or a medical specialist.

6.4 Data Safety Monitoring Board (DSMB)

An independent data monitoring committee will perform the interim analysis and decide whether the trial should be stopped. All members of this committee should be excluded from the trial. The statistical analysis will not be disclosed to anyone who is not a member of this committee

7. STATISTICAL ANALYSIS

7.1 Descriptive statistics

The primary endpoint occurrence of delirium within 10 days after surgery will be analyzed using the Chi-square test. Delirium will be presented as qualitative data. The secondary endpoints will be analyzed using a student-T test. These analyses will be one sided. Further analyses will be performed by logistic regression using the occurrence of delirium as dependent variable.

8. ETHICAL CONSIDERATIONS

8.1 Regulation statement

All patients will be informed of the aims of the study, the procedures and the mechanism of treatment allocation. They will be informed as to the strict confidentiality of their patient data, but that their medical records may be reviewed for trial purposes by authorized individuals other than their treating physician.
It will be emphasized that the participation is voluntary and that the patient is allowed to refuse further participation in the protocol whenever he/she wants. This will not prejudice the patient's subsequent care. Documented informed consent must be obtained for all patients included in the study before they are registered in the study.
This study is conducted in agreement with the Declaration of Helsinki (Tokyo, Venice, Hong Kong, Somerset West and Edinburgh amendments) and in accordance with the Medical Research Involving Human Subjects Act (WMO) and other guidelines, regulations and Acts

According to the ICH guidelines on Good Clinical Practice and Dutch law, the written informed consent form should be signed and personally dated by the patient and the responsible investigator.

8.2 Recruitment and consent

Patients will be informed on the study by a research nurse at the outpatient clinic or 24 hours prior to surgery in case of "acute" admissions. The research nurse will give the patient the information letter and they will be given up to 24 hours prior to surgery to consider their decision. Upon consent the patient and the research nurse will sign the informed consent form. The patient information letter and informed consent form are attached as a separate document.

8.3 Benefits and risks assessment, group relatedness

Delirium is a highly prevalent and potential lethal postoperative complication in elderly patients. The literature suggests that perioperative intervention by a geriatric team decreases the incidence of this complication. The intervention by the geriatric team entails adjustments in the daily routine of the patient, the diet and medication prescribed but no additional invasive of uncomfortable procedures. The intervention is tailored to make the patient as comfortable as possible. We therefore think it is beneficial to expose the participating subjects to extra questionnaires perioperatively that will take up some of their time as this is the only way for us to show the benefit of the geriatric intervention. This study has to be performed in the fragile elderly patients as they are the patients most likely to develop postoperative delirium and most likely to benefit from the intervention.

8.4 Compensation for injury

The investigators wish to obtain dispensation from the insurance. This has been requested from the METC.

9. ADMINISTRATIVE ASPECTS AND PUBLICATION

9.1 Handling and storage of data and documents

All data will be handled confidentially and anonymously. A subject identification code list will be used to link the data to the subject. The code will not be based on the patients intials and birth-date. The key to the code will be safeguarded by the investigator. The handling of personal data will comply with the Dutch Personal Data Protection Act.

9.2 Amendments

All amendments will be notified to the METC that gave a favourable opinion.

9.3 Annual progress report

The sponsor/investigator will submit a summary of the progress of the trial to the accredited METC once a year. Information will be provided on the date of inclusion of the first subject, numbers of subjects included and numbers of subjects that have completed the trial, serious adverse events/ serious adverse reactions, other problems, and amendments.

9.4 End of study report

The investigator [sponsor] will notify the accredited METC [and the competent authority] of the end of the study within a period of 8 weeks [90 days]. The end of the study is defined as 3 months postoperative for the last patient included.

In case the study is ended prematurely, the investigator [sponsor] will notify the accredited METC [and the competent authority within 15 days], including the reasons for the premature termination.

Within one year after the end of the study, the investigator/sponsor will submit a final study report with the results of the study, including any publications/abstracts of the study,

to the accredited METC [and the Competent Authority].

9.5 Public disclosure and publication policy

The research data will be publicly disclosed and published independent of the outcome of this study. Members of the writing committee including the principal investigators, will all participate in the publication process.

10. REFERENCES

- Audisio RA, Bozzetti F, et al. The surgical management of elderly cancer patients recommendations of the SIOG surgical task force. Eur J Cancer, 2004; 40: 926-38
- Brennan T.A., Leape, L.L., Laird, N.M., et al). Incidence of adverse events and negligence in
- hospitalized patients: Results of the Harvard Medical Practice Study. N Engl J Med, 1991;324, 370-376.
- Damhuis R.A.M, Meurs C.J.C, Meijer W. Postoperative mortality after cancer surgery in octogenarians and nonagenarians; results from a series of 5,390 patients. World J Surg Oncol, 2005; 3:71.
- Dijkstra et al. Het meten van zorgafhankelijkheid met de Zorgafhankelijkheidsschaal (ZAS): een handleiding. 1999. Groningen. Noordelijk Centrum voor Gezondheidsvraagstukken. ISBN 90-72156-46-3.
- Fentiman I.S, Are the elderly receiving appropriate therapy for cancer?. Ann. Oncol. 1996; 7: 657-58
- Francis J., Martin, D., Kapoor, W.N. A prospective study of delirium in hospitalized elderly. JAMA, 1990;263, p. 1097-1101.
- Franco K., Litaker D., Locala J. Bronson D. The Cost of delirium in the Surgical Patient.
- Psychosomatics, 2001; 42:1, 68-73.
- Frieswijk N., Buunk A.P., Steverink N., Slaets J.P.J. The interpretation of Social Comparison and Its Relation to Life Satisfaction Among Elderly People: Does Frailty Make a Difference? J Gerontol B Psychol Sci Soc Sci ,2004; 59B;5:P250-P257.
- Hoekstra HJ. Cancer surgery in the elderly Eur J Cancer. 2001; 37 (7):235-44
- Inouye S.K. Bogardus S.T., Charpentier P.A,, Leo-Summers L,Campora D.A, Holford T.A, Cooney L.M. A multicomponent intervention to prevent delirium in hospitalized older patients. N Engl J Med , 1999; 340; 9, 669-676
- Kalisvaart K.J., de Jonghe JF, Bogaards MJ, Vreeswijk R, Egberts TC, Burger BJ, Eikelenboom P, van Gool WA. Haloperidol prophylaxis for elderly hip-surgery patients at risk for delirium: a randomized placebo-controlled study. J Am Geriatr Soc, 2005;53(10):1658-66.
- Kalisvaart. Primary prevention of delirium in the elderly. Thesis 2005
- Marcantonio ER, Flacker JM, Wright RJ, Resnick NM.Reducing delirium after hip fracture: a randomized trial.J Am Geriatr Soc. 2001 May;49(5):516-22.
- Mast van der R.C., Huyse FJ, Drooglever H.A. et al. Dutch Institute for Healthcare Improvement (CBO) Guideline Delirium. 2004, Boom Amsterdam..
- Olde Rikkert, M. In: Notitie Werkgroep Ouderenzorg t.b.v. ZonMw deelprogramma Klinische Geriatrie. Ouderen nu en in de toekomst. Gezondheid, verpleging en verzorging 2000-2020. 2004; RIVM 270502001.
- Repetto L., Ventrino A, Fratino L. et al. Geriatric oncology: a clinical approach to the older patient with cancer. Eur J Cancer, 2003; 39 (7): 870-880.
- Rothschild, J.M., Bates, D.W., Leape, L.L. Preventable medical injuries in older patients. Arch Intern Med, 2000; 160, 2717-2728.
- Sager, M.A., Rudberg, M.A., Jalaluddin, M. et al.). Promoting well-being in frail elderly people: Theory and intervention.Dissertation, 2004, Groningen University.
- Schuurmans MJ, Shortridge-Bagget LM, Duursma SA. The Delirium Observation Screening Scale: a screening instrument for delirium. Res Theory Nurs Pract. 2003 17(1):31-50
- Schuurmans, H., Steverink, N., Lindenberg, S., Frieswijk, N., Slaets, J.P.J.. Old or frail: what tells us more? J Gerontol A Biol Sci Med Sci. 2004 Sep;59(9):M962-5.
- Slaets JP. [The importance of the 'frailty-concept' in geriatric assessment]. Tijdschr.Gerontol.Geriatr.1998;29:276-8.

- Slaets JPJ. De oudere patiënt. In Consultatieve inwendige geneeskunde. ROB Gans, SJ Hoorntje en RJM Strack van Schijndel (red). Bohn Stafleu van Loghum, Houten 2004.
- Slaets, J.P.J., Kauffmann, R.H., Duivenvoorden, H.J., Pelemans, W., Schudel, W.J.). A randomized trial of geriatric liaison intervention in elderly medical inpatients. Psychosom Med. 1997 Nov-Dec;59(6):585-91.
- Steverink, N., Slaets, J.P.J., Schuurmans, H., Lis, M. van. Measuring frailty: development and testing of the Groningen Frailty Indicator (GFI). Gerontologist, 2001; 41SI, 236-237.
- Trzepazc P.T., Mulsant, B.H.,Amanda-Dew, M., Pasternak, R.,Sweet, R.A. et al. Is delirium different when it occurs in dementia? A study using the delirium rating scale. J Neuropsychiatry Clin Neurosci. 1998 Spring;10(2):199-204.
- Van Hemert A.M., Van der Mast RC, Hengeveld MW et al. Excess mortality in general hospital patients with delirium: a 5 year follow up of 519 patients seen in psychiatric consultation. J Psychosom Res, 1994;38: 339-346.
- Vercelli M, Capocaccia D, Quaglia A, Casella C, Puppo A, Coebergh J W W and EUROCARE Working Group. Relative survival in elderly European cancer patients: evidence for health care inequalities. Crit Rev Oncol Hematol, 2000; 35: 161-79
- Wymenga AN, Slaets JP, Sleijfer DT. Treatment of cancer in old age, shortcomings and challenges. Neth.J.Med. 2001;59:259-66

Informed consent. Patiënten-informatiebrief behorend bij

Onderzoek naar postoperatief functioneren van kwetsbare ouderen

Geachte heer/mevrouw,

Binnenkort ondergaat u een operatie.
U zult rondom deze operatie enkele dagen tot weken opgenomen zijn op de afdeling chirurgie of oncologie van dit ziekenhuis. Wij onderzoeken op dit moment in dit ziekenhuis hoe patiënten ouder dan 65 jaar herstellen van een operatie. Wij zijn daarbij voornamelijk geïnteresseerd in het beloop na de operatie en in hun functioneren in de eerste 3 maanden na de operatie. Wij onderzoeken mogelijkheden om de zorg rondom de operatie te verbeteren en daarmee het beloop na de operatie zo gunstig mogelijk te laten verlopen. Wij willen u daarom vragen deel te nemen aan dit onderzoek.

Als u besluit aan dit onderzoek deel te nemen houdt dit voor u het volgende in:
Een verpleegkundige zal u een aantal vragen stellen met betrekking tot uw gezondheid en woonomstandigheden op dit moment. Dit neemt ongeveer 15 minuten in beslag. Aan de hand van de uitkomsten zal een inschatting worden gemaakt hoe groot de kans is dat de operatie en de ziekenhuisopname van invloed kunnen zijn op uw functioneren in de eerste 3 maanden na de operatie. Dit bepaalt mede of u verder deel zal nemen aan dit onderzoek.
Het is mogelijk dat bij deelname aan dit onderzoek een geriater (internist gespecialiseerd in de zorg van de oudere patiënt) en verpleegkundig specialist betrokken wordt bij uw behandeling tijdens uw opname in het ziekenhuis. Het al dan niet betrekken van deze mensen bij uw behandeling wordt door het loting bepaald. Aan alle patiënten die meedoen aan het onderzoek zullen tijdens de opname dagelijks enkele vragen worden gesteld met betrekking tot het functioneren en het welbevinden op dat moment. Dat neemt niet meer dan enkele minuten van uw tijd in beslag.
Bij ontslag uit het ziekenhuis en 3 maanden na de operatie zullen dezelfde vragen met betrekking tot gezondheid, functioneren en woonomstandigheden opnieuw worden gesteld. Het beantwoorden van deze vragen zal ongeveer 30 tot 60 minuten van uw tijd vergen.

Bij het onderzoek zullen gegevens uit uw medisch dossier worden gebruikt. Artsen, verpleegkundigen en onderzoekers betrokken bij dit onderzoek zullen toegang hebben tot deze gegevens. Bij de analyse van deze gegevens blijft uw privacy gewaarborgd.

Aan deelname zijn geen risico's verbonden en uw operatieve behandeling wordt hierdoor niet beïnvloed. Het staat u op ieder ogenblik vrij om deelname aan dit onderzoek te weigeren. Dit heeft geen consequenties voor uw verdere behandeling. Ook bent u volledig vrij om zonder opgave van redenen op ieder moment uw deelname aan het onderzoek te beëindigen. Ook dit heeft geen enkel effect op de wijze waarop wij u verder zullen behandelen.

Een onafhankelijke arts (niet betrokken bij dit onderzoek) kan u bereiken voor eventuele vragen: Dr. J.T.M. Plukker, afdeling Heelkunde, UMCG, tel: 050-3616161

Bij voorbaat dank voor uw medewerking,
Namens het onderzoeksteam
Prof. Dr. J.P.J. Slaets
Hoofd Academisch Centrum Ouderengeneeskunde
UMCG

Toestemmingsformulier behorend bij

Onderzoek naar postoperatief functioneren van kwetsbare ouderen

Ik stem erin toe deel te nemen aan bovengenoemd onderzoek. Door loting wordt bepaald of een geriater en verpleegkundig specialist betrokken worden bij mijn behandeling tijdens de ziekenhuisopname.

Tijdens dit onderzoek zal ik een aantal keren vragen beantwoorden met betrekking tot mijn gezondheid, functioneren en woonomstandigheden. De mensen betrokken bij dit onderzoek (artsen, verpleegkundigen, onderzoekers) hebben toegang tot mijn medisch dossier. Resultaten van dit onderzoek zullen worden gepubliceerd in medische tijdschriften waarbij mijn privacy gewaarborgd blijft.

Aan deelname zijn geen risico's verbonden en mijn operatieve behandeling wordt hierdoor niet beïnvloed. Het staat mij op ieder ogenblik vrij om deelname aan dit onderzoek te weigeren. Dit heeft geen consequenties voor verdere behandeling. Ook ben ik volledig vrij om zonder opgave van redenen op ieder moment deelname aan het onderzoek te beëindigen. Ook dit heeft geen enkel effect op de wijze waarop ik verder zal worden behandeld.

Handtekening Patiënt

Handtekening lid onderzoeksteam

Naam Patiënt

Naam lid onderzoeksteam

Datum:

Ziekenhuis

Groningen Frailty Index

Mobiliteit

Kan patiënt zonder enige hulp van iemand anders zelfstandig deze taak uitvoeren? (gebruik maken van hulpmiddelen als stok, rolator, rolstoel, geldt als zelfstandig

1. Boodschappen doen JA/NEE
2. Buitenshuis rondlopen (rondom huis of naar de buren) JA/NEE
3. Aan- en uitkleden JA/NEE
4. Toiletbezoek JA/NEE

Lichamelijke fitheid

5. Welk rapportcijfer geeft patiënt zichzelf voor lichamelijke fitheid? (0-10, 0 is erg slecht, 10 is erg goed)

Visus

6. Ondervindt patiënt problemen in dagelijks leven door slecht zien? JA/NEE

Gehoor

7. Ondervindt patiënt problemen in het dagelijks leven door slecht horen?
   JA/NEE

Voeding

8. Is patiënt afgelopen 6 maanden veel (6kg) afgevallen zonder dit zelf te willen? (of 3 kg in een maand)
   JA/NEE

Co-morbiditeit

9. Gebruikt patiënt momenteel 4 of meer verschillende soorten medicijnen? JA/NEE

Cognitie

10. Heeft patiënt momenteel klachten over zijn geheugen (of bekend met dementie)
    JA/NEE

Psychosociaal

11. Ervaart patiënt wel eens een leegte om zich heen? JA/NEE
12. Mist patiënt wel eens mensen om zich heen? JA/NEE
13. Voelt patiënt zich wel eens in de steek gelaten?
    JA/NEE
14. Heeft patiënt zich de laatste tijd somber of neerslachtig gevoeld? JA/NEE
15. Heeft patiënt zich de laatste tijd nerveus of angstig gevoeld? JA/NEE

Scoring GFI:

Vraag 1t/m 4: zelfstandig = 0, niet zelfstandig = 1

Vraag 5: 0-6= 1, 7-10= 0

Vraag 6 t/m 9; nee = 0, ja = 1

Vraag 10: nee = 0, soms = 0, ja = 1

Vraag 11 t/m 15 nee= 0, soms = 1, ja= 1

CARE DEPENDENCY SCORE

CDS-vpl *1*

Beoordeel de patiënt, die aan uw zorg is toevertrouwd, op elk van de 15 kenmerken van de zorgafhankelijkheidsschaal. Geef aan welke beschrijving het beste van toepassing is. Ga uit van
de mogelijkheden die de patiënt bezit en niet zozeer welke handelingen de verzorgende of verpleegkundige overneemt.

Voorbeeld:

1 Eten en drinken **De mate waarin de aan uw zorg toevertrouwde patiënt in staat is zelfstandig te voldoen aan de behoefte aan eten en drinken**

1 Volledig zorgafhankelijk
2 In grote mate zorgafhankelijk
3 Gedeeltelijk zorgafhankelijk
4 Beperkt zorgafhankelijk
5 Vrijwel zelfstandig

Geef van alle 15 kenmerken een beoordeling, sla geen kenmerk over. Ten slotte wordt u gevraagd in vraag 16 aan te geven welke omschrijving van zorgafhankelijkheid van toepassing is op de patiënt als geheel. Probeer bij twijfel tussen 2 beschrijvingen tot één keuze te komen, door in te schatten welke mogelijkheid de betreffende patiënt bezit.
Er zijn 15 kenmerken met ieder 5 criteria van zorgafhankelijkheid opgenomen in de zorgafhankelijkheidsschaal. OMCIRKEL het CIJFER van het criterium welke het beste bij de betreffende patiënt past. (Maak uw keuze door één van de cijfers te omcirkelen)

1 Eten en drinken **De mate waarin de patiënt in staat is zelfstandig te voldoen aan de behoefte aan eten en drinken**

1 Volledig zorgafhankelijk
2 In grote mate zorgafhankelijk
3 Gedeeltelijk zorgafhankelijk
4 Beperkt zorgafhankelijk
5 Vrijwel zelfstandig

2 Incontinentie **De mate waarin de patiënt het vermogen heeft de uitscheiding van urine en faeces willekeurig te beheersen**

1 Volledig zorgafhankelijk
2 In grote mate zorgafhankelijk
3 Gedeeltelijk zorgafhankelijk
4 Beperkt zorgafhankelijk
5 Vrijwel zelfstandig

3 Lichaamshouding **De mate waarin de patiënt in staat is bij een bepaalde activiteit een juiste houding aan te nemen**

1 Volledig zorgafhankelijk
2 In grote mate zorgafhankelijk
3 Gedeeltelijk zorgafhankelijk
4 Beperkt zorgafhankelijk
5 Vrijwel zelfstandig

4 Mobiliteit **De mate waarin de patiënt fysiek in staat is zich zelfstandig voort te bewegen**

1 Volledig zorgafhankelijk
2 In grote mate zorgafhankelijk
3 Gedeeltelijk zorgafhankelijk
4 Beperkt zorgafhankelijk
5 Vrijwel zelfstandig

5 Dag-nachtritme **De aard van het slaap-waakpatroon (bio-ritme) van de patiënt**

1 Volledig zorgafhankelijk
2 In grote mate zorgafhankelijk
3 Gedeeltelijk zorgafhankelijk
4 Beperkt zorgafhankelijk
5 Vrijwel zelfstandig

6 Aan- en uitkleden **De mate waarin de patiënt beschikt over vaardigheden om zich zelfstandig aan- en uit te kleden**

1 Volledig zorgafhankelijk

2 In grote mate zorgafhankelijk
3 Gedeeltelijk zorgafhankelijk
4 Beperkt zorgafhankelijk
5 Vrijwel zelfstandig

7 Lichaamstemperatuur **De mate waarin de patiënt in staat is zelfstandig de lichaamstemperatuur te beschermen tegen externe invloeden**

1 Volledig zorgafhankelijk
2 In grote mate zorgafhankelijk
3 Gedeeltelijk zorgafhankelijk
4 Beperkt zorgafhankelijk
5 Vrijwel zelfstandig

8 Hygiëne **De mate waarin de patiënt in staat is zelfstandig zorg te dragen voor zijn/haar lichaamsverzorging**

1 Volledig zorgafhankelijk
2 In grote mate zorgafhankelijk
3 Gedeeltelijk zorgafhankelijk
4 Beperkt zorgafhankelijk
5 Vrijwel zelfstandig

9 Vermijden van gevaar **De mate waarin de patiënt in staat is zelfstandig voor zijn/haar eigen veiligheid te zorgen**

1 Volledig zorgafhankelijk
2 In grote mate zorgafhankelijk
3 Gedeeltelijk zorgafhankelijk
4 Beperkt zorgafhankelijk
5 Vrijwel zelfstandig

10 Communicatie **De mate waarin de patiënt in staat is te communiceren**

1 Volledig zorgafhankelijk
2 In grote mate zorgafhankelijk
3 Gedeeltelijk zorgafhankelijk
4 Beperkt zorgafhankelijk
5 Vrijwel zelfstandig

11 Contact met anderen **De mate waarin de patiënt in staat is tot het aangaan, onderhouden en afbreken van sociaal contact**

1 Volledig zorgafhankelijk
2 In grote mate zorgafhankelijk
3 Gedeeltelijk zorgafhankelijk
4 Beperkt zorgafhankelijk
5 Vrijwel zelfstandig

12 Waarde- en normbesef **De mate waarin de patiënt in staat is zelfstandig leefregels te hanteren**

1 Volledig zorgafhankelijk
2 In grote mate zorgafhankelijk
3 Gedeeltelijk zorgafhankelijk
4 Beperkt zorgafhankelijk
5 Vrijwel zelfstandig

13 Dagelijkse activiteiten **De mate waarin de patiënt in staat is zelfstandig invulling te geven aan de dagelijkse bezigheden binnen de woongroep**

1 Volledig zorgafhankelijk
2 In grote mate zorgafhankelijk
3 Gedeeltelijk zorgafhankelijk
4 Beperkt zorgafhankelijk
5 Vrijwel zelfstandig

14 Recreatieve activiteiten **De mate waarin de patiënt in staat is zelfstandig deel te nemen aan ontspannende activiteiten buiten de woongroep**

1 Volledig zorgafhankelijk
2 In grote mate zorgafhankelijk
3 Gedeeltelijk zorgafhankelijk
4 Beperkt zorgafhankelijk
5 Vrijwel zelfstandig

15 Leervermogen **De mate waarin de patiënt in staat is om zelfstandig kennis en/of vaardigheden aan te leren dan wel het geleerde in stand te houden**

1 Volledig zorgafhankelijk
2 In grote mate zorgafhankelijk
3 Gedeeltelijk zorgafhankelijk
4 Beperkt zorgafhankelijk
5 Vrijwel zelfstandig

16 Samenvatting **Geef aan welke omschrijving van zorgafhankelijkheid van toepassing is op de patiënt**

1 Volledig zorgafhankelijk
2 In grote mate zorgafhankelijk
3 Gedeeltelijk zorgafhankelijk
4 Beperkt zorgafhankelijk
5 Vrijwel zelfstandig

- de **DOS Schaal eindscore** wordt berekend door de totaal score deze dag te delen door 3; de DOS eindscore ligt tussen de 0 en 13
- een DOS Schaal eindscore < 3 betekent dat de patiënt waarschijnlijk niet delirant is, een DOS Schaal eindscore ≥ 3 betekent dat de patiënt waarschijnlijk wel delirant is*

DELIRIUM RATING SCALE-R-98

(DRS-R-9S)

Vertaling: H.N. Sno, A.C. van der Mast.

Dit is een herziene versie van de Delirium Rating Scale (Trzepacz et al. 1988). Deze wordt gebruikt voor een eerste onderzoek en herhaalde metingen van de ernst van delirante symptomen. De som van de dertien itemscores is een ernstscore. In aanvulling op het psychiatrisch onderzoek van de patiënt worden alle beschikbare bronnen van informatie gebruikt bij het scoren van de items (verpleegkundigen, familie, status). Bij een reeks herhaalde metingen van de ernst van het delirium, dienen tussen de metingen redelijke tijdsintervallen gekozen te worden om veranderingen van betekenis vast te leggen aangezien de ernst van de delirante symptomen zonder interventies kan wisselen.

DRS-R-98 Ernst-schaal

1 Stoornis van het slaap-waakritme
Maak bij het scoren van het slaap-waakpatroon gebruik van alle informatiebronnen, zoals van familie, hulpverleners, verpleegkundige rapporten en de patiënt. Probeer een onderscheid te maken tussen slapen en rusten met gesloten ogen.

0 Niet aanwezig
1 Lichte verstoring van het 's nachts doorslapen of zo nu en dan slaperigheid overdag
2 Matig ernstige verstoring van het slaap-waakritme (bijvoorbeeld: *valt* in slaap tijdens gesprekken, doet overdag dutjes of wordt 's nachts meerdere malen kortdurend wakker, gepaard gaande met verwardheid/gedragsveranderingen of slaapt 's nachts heel weinig
3 Ernstige verstoring van slaap-waakritme (bijvoorbeeld omkering van het dag-nachtritme) of ernstige circadiane fragmentatie gepaard gaand met meerdere periodes van slapen en waken of ernstige slapeloosheid

2 Waarnemingsstoornissen en hallucinaties
Illusionaire vervalsingen en hallucinaties kunnen iedere zintuiglijke modaliteit betreffen. Waarnemingsstoornissen zijn 'eenvoudig' indien deze ongecompliceerd zijn, zoals geluid, lawaai, kleur, vlekken of flitsen en 'complex' wanneer deze multidimensioneel zijn, zoals stemmen, muziek, mensen, dieren of voorvallen. Scoor op basis van de anamnese van de patiënt, van de verzorger of van observatie.

0 Niet aanwezig
1 Lichte waarnemingsstoornissen (bijvoorbeeld gevoelens van derealisatie of depersonalisatie; of de patiënt is niet in staat om een onderscheid te maken tussen dromen en werkelijkheid)
2 Aanwezigheid van illusionaire vervalsingen
3 Aanwezigheid van hallucinaties

3 Wanen
Wanen kunnen ieder type zijn, maar betreffen meestal paranoïde wanen. Scoor op basis van anamnese van de patiënt, familie of verzorger. Scoor als waan indien het onwaarschijnlijk is dat de ideeën waar zijn, terwijl de patiënt erin gelooft en door logische argumenten niet op andere gedachten gebracht kan worden. Waanideeën kunnen niet anderszins verklaard worden door de culturele of religieuze achtergrond van de patiënt.

0 Niet aanwezig
1 Licht achterdochtig, zeer waakzaam of in beslag genomen
2 Ongewone en overwaardige ideeën die niet het niveau van een waan bereiken en waar zouden kunnen zijn
3 Wanen

4 Affectlabiliteit
Scoor het affect van de patiënt op basis van zichtbare uitingen van emoties en niet op basis van een beschrijving van wat de patiënt voelt.

0 Niet aanwezig
1 Het affect is enigszins veranderd of past niet helemaal bij de situatie; kan wisselen in verloop van uren: patiënten hebben emoties meestal onder controle
2 Het affect past vaak niet bij de situatie en kan wisselen in verloop van minuten; patiënten hebben meestal geen controle over emoties, hoewel ze wel reageren op correcties van anderen
3 Ernstige en aanhoudende emotionele ontremming; het affect wisselt snel, is niet passend bij de situatie en reageert niet op correcties van anderen

5 Taal
Scoor afwijkingen van gesproken, geschreven of gebarentaal, die niet toegeschreven kunnen worden aan dialect of stotteren. Let op tempo en intonatie, grammatica, begrip, semantische inhoud en benoemen. Test het begrip en benoemen zo nodig non-verbaal door de patiënt opdrachten te laten vervullen of te laten aanwijzen.

0 Normaal taalgebruik
1 Lichte verslechtering zoals woordvindingsstoornissen of problemen met benoemen, tempo of intonatie.
2 Matig ernstige verslechtering zoals moeite met begrijpen of een tekortschietende zinvolle communicatie (semantische inhoud)
3 Ernstige verslechtering zoals betekenisloze semantische inhoud, woordsalade, mutisme of ernstig verminderd begrip

6 Afwijkingen van het denkproces
Baseer score van afwijkingen van denkprocessen op verbale en geschreven uitingen. Scoor dit item niet, indien een patiënt niet kan spreken of schrijven.

0 Normale denkprocessen
1 Tangentieel of circumstantieel
2 Verbanden zijn nu en dan onsamenhangend, maar merendeels te begrijpen
3 Verbanden zijn meestal onsamenhangend

7 Motorische agitatie
Scoor op basis van eigen observatie en die van anderen, zoals bezoek, familie en afdelingsstaf. Laat dyskinesie, tics of chorea buiten beschouwing.

0 Geen onrust of agitatie
1 Lichte onrust van grove motoriek of lichte plukkerigheid
2 Matig ernstige motorische agitatie, zoals duidelijke bewegingsonrust van de extremiteiten, rusteloos op en neer lopen, plukkerigheid, uittrekken van infuuslijnen enzovoort
3 Ernstige motorische agitatie, zoals motorische agressie of noodzaak van dwangmiddelen of separeren

8 Motorische remming
Scoor bewegingen op basis van directe observatie of observaties van anderen zoals familie, bezoek of afdelingsstaf. Laat remming als gevolg van parkinsonisme buiten beschouwing, evenals slaperigheid of slapen.

0 Geen traagheid van willekeurige bewegingen
1 Licht verminderde frequentie, spontaniteit of snelheid van de motoriek zodanig dat het enigszins interfereert met het onderzoek
2 Matige ernstig verminderde frequentie, spontaniteit of snelheid van motoriek zodanig dat het interfereert met deelname aan activiteiten of de zelfverzorging
3 Ernstige motorische remming met weinig spontane bewegingen

9 Oriëntatie
Aan patiënten die niet kunnen spreken kunnen visueel of auditief multiple-choice-antwoorden aangeboden worden. Patiënten die langer dan drie weken zijn opgenomen mogen er maximaal zeven dagen in plaats van twee dagen naast zitten. Desoriëntatie in persoon betekent het niet herkennen van bekende personen en is ongestoord bij het niet op de naam kunnen komen, terwijl er

wel sprake is van herkenning. De meest ernstige vorm van desoriëntatie in persoon is het niet kennen van de eigen identiteit en is zeldzaam. Desoriëntatie in persoon treedt meestal op na desoriëntatie in tijd en/of persoon.

0 Georiënteerd in persoon, plaats en tijd
1 Desoriëntatie in tijd (bijvoorbeeld meer dan twee dagen, of verkeerde maand of verkeerd jaar) of plaats (bijvoorbeeld gebouw, stad, provincie), maar niet beide
2 Desoriëntatie in tijd en plaats
3 Desoriëntatie in persoon

10 Aandachtsconcentratie
Bij patiënten met zintuiglijke beperkingen of die geïntubeerd zijn of hun handen niet kunnen gebruiken dient een andere modaliteit dan schrijven te worden getest. De aandachtsconcentratie kan onderzocht worden tijdens het interview (bijvoorbeeld verbale perseveraties, afleidbaarheid en moeite met wisselen van de aandacht) en/of door het gebruiken van specifieke tests, bijvoorbeeld digit-scan.

0 Alert en oplettend
1 Licht afleidbaar of lichte moeite om de aandacht vast te houden, maar in staat zich te herstellen met behulp van aanwijzingen. Bij formeel testen worden slechts geringe fouten gemaakt en de patiënt reageert niet significant trager.
2 Matig ernstige aandachtsconcentratiestoornis met moeite aandacht te richten en vast te houden. Bij formeel testen maakt de patiënt verschillende fouten en heeft aansporing nodig om de aandacht te richten of de taak te voltooien.
3 Ernstige problemen met het richten en/of vasthouden van de aandacht, met veel onjuiste of incomplete antwoorden of onvermogen opdrachten op te volgen. Afleidbaar door andere geluiden of dingen die gebeuren in de omgeving.

11 Kortetermijngeheugen
Gedefinieerd als het herinneren van informatie (bijvoorbeeld drie items die ofwel verbaal of visueel worden aangeboden) na een interval van ongeveer twee tot drie minuten. Wanneer formeel getest wordt, dient de informatie eerst adequaat geregistreerd te zijn alvorens het geheugen getest wordt. Zowel het aantal pogingen om te registreren als het *effect* van het geven van een hint kan op het scoreformulier worden genoteerd. Het is patiënten niet toegestaan om oefenen gedurende de intervalperiode en zij gedurende die tijd worden afgeleid. De patiënt mag de onderzoeker pratend of non-verbaal de identiteit van de juiste items laten weten. Gebreken in het kortetermijngeheugen die opgevallen zijn tijdens het interview kunnen ook worden gebruikt.

0 Kortetermijngeheugen intact
1 Herinnert 2/3 items; zou zich het derde item herinneren nadat een aanwijzing betreffende de categorie is gegeven
2 Herinnert 1/3 items; zou zich andere items herinneren nadat aanwijzingen betreffende de categorie is gegeven
3 Herinnert 0/3 items

12 Langetermijngeheugen
Kan formeel onderzocht worden of door middel van een interview over herinnering van informatie omtrent persoonlijk verleden (bijvoorbeeld medische voorgeschiedenis of informatie of ervaringen die afgeleid kunnen worden via een andere bron) of algemene informatie die cultureel relevant is. Wanneer formeel getest wordt, gebruik een verbale en/of visuele modaliteit voor drie items die adequaat geregistreerd en gereproduceerd worden na minimaal vijf minuten. De patiënt dient niet de gelegenheid te krijgen om te repeteren gedurende de intervalperiode bij een formele test.
Houd, wat betreft vragen omtrent algemene informatie, rekening met patiënten die de lagere school niet hebben afgemaakt of die mentaal geretardeerd zijn. Het scoren van de ernst van de gebreken kan een oordeel inhouden over alle methoden waarop het langetermijngeheugen is onderzocht, inclusief de capaciteit van het langetermijngeheugen betreffende het recente en/of nabije verleden die gedurende het interview informeel is getest alsmede elke formele test van het langetermijngeheugen betreffende het recente verleden waarbij gebruik is gemaakt van drie items.

0 Geen significante stoornis van het langetermijngeheugen
1 Herinnert 2/3 items en/of heeft geringe moeite met het herinneren van details van andere langetermijn informatie
2 Herinnert 1/3 items en/of heeft matig ernstige moeite met het herinneren van andere langetermijninformatie
3 Herinnert 0/3 items en/of heeft zeer ernstige moeite met het herinneren van details

van andere langetermijninformatie

13 Visuospatieel oriëntatievermogen
Onderzoek informeel en formeel. Let op de problemen van de patiënt met het vinden van de weg in leefgebieden of omgeving (bijvoorbeeld verdwalen). Test formeel door tekenen en natekenen van een ontwerp, het rangschikken van puzzelstukjes, of door het tekenen van een kaart en het benoemen van belangrijke steden enzovoort. Houd rekening met ieder visueel gebrek dat het uitvoeren kan beïnvloeden.

0 Geen vermindering
1 Lichte vermindering zodat het globale ontwerp, de meeste details correct zijn; en/of weinig moeite om de weg te vinden in zijn/haar omgeving
2 Matig ernstige vermindering met verstoorde inschatting van het globale ontwerp en/of diverse fouten van details of onderdelen; en/of moet herhaald op de juiste richting gewezen worden om verdwalen te voorkomen in een nieuwere omgeving ondanks moeite om bekende objecten te lokaliseren in de directe omgeving
3 Zeer ernstige vermindering bij formeel testen; en/of herhaald zwerven of verdwalen in de omgeving

DRS-R-98 facultatieve diagnostische items
De volgende drie items kunnen worden gebruikt als steun bij de differentiatie van het delirium van andere stoornissen voor diagnostische en wetenschappelijke doeleinden.

Chronologisch begin van de symptomen
Scoor de abruptheid van het begin van de eerste symptomen van de stoornis of episode die op dit moment onderzocht wordt, niet de totale duur van de symptomen. Let hierbij uitsluitend op het begin van symptomen die toegeschreven worden aan een delirium, wanneer het delirium tegelijkertijd optreedt met een andere preëxistente psychiatrische stoornis. Bijvoorbeeld, wanneer de score wordt bepaald tijdens een delirante episode als gevolg van een overdosis bij een patiënt met een depressieve episode, scoor dan het begin van de delirante symptomen.

0 Geen significante verandering van het normale of lang bestaand standaard gedrag
1 Geleidelijk begin van symptomen, in het verloop van een periode van enkele weken tot een maand
2 Acute verandering van gedrag of persoonlijkheid, in het verloop van dagen tot een week
3 Abrupte verandering van gedrag, in het verloop van enkele uren tot een dag

Wisselende ernst van symptomen
Scoor het verergeren en verminderen van een enkel symptoom of een symptomencomplex gedurende het tijdsbestek waarop het onderzoek gericht is, Betreft meestal cognitie, affect, intensiteit van hallucinaties, denkstoornis of taalstoornis, Bedenk dat waarnemingsstoornissen meestal intermitterend optreden, maar kunnen samenkomen in een periode van grotere intensiteit, wanneer andere symptomen in ernst fluctueren,

0 Geen wisseling van ernstsymptomen
1 Intensiteit van symptomen wisselt wat betreft ernst in uren
2 Intensiteit van symptomen wisselt wat betreft ernst in minuten

Lichamelijke aandoening
Scoor de mate waarin een fysiologisch, somatisch of farmacologisch probleem specifiek aangewezen kan worden als oorzaak voor de symptomen, die onderzocht worden, Veel patiënten hebben zulke problemen, maar ze kunnen al of niet een causale relatie hebben met de symptomen die gescoord worden,

0 Niet aanwezig of actief
1 Aanwezigheid van een lichamelijke aandoening die de psychische toestand kan beïnvloeden
2 Geneesmiddel, infectie, metabole stoornis, CZS-laesie of ander somatisch probleem dat specifiek aangewezen kan worden als oorzaak voor het veranderd gedrag of psychische toestand,

SF-36

Hoe beoordeelt u uw gezondheid? (RAND-SF-36)

In deze vragenlijst wordt naar uw gezondheid gevraagd. Wilt u elke vraag beantwoorden door het juiste hokje aan te kruisen. Wanneer u twijfelt over het antwoord op een vraag, probeer dan het antwoord te geven dat het meest van toepassing is.

1 Wat vindt u, over het algemeen genomen, van uw gezondheid?

- ☐ uitstekend
- ☐ zeer goed
- ☐ goed
- ☐ matig
- ☐ slecht

2 *In vergelijking met een jaar geleden*, hoe zou u *nu* uw gezondheid in het algemeen beoordelen?

- ☐ veel beter dan een jaar geleden
- ☐ iets beter dan een jaar geleden
- ☐ ongeveer hetzelfde als een jaar geleden
- ☐ iets slechter dan een jaar geleden
- ☐ veel slechter dan een jaar geleden

3 De volgende vragen gaan over dagelijkse bezigheden. Wordt u door **uw gezondheid** op dit moment beperkt bij deze bezigheden? Zo ja, in welke mate?

		**ja, ernstig beperkt**	**ja, een beetje beperkt**	**nee, helemaal niet beperkt**
a	*forse inspanning* zoals hardlopen, zware voorwerpen tillen, inspannend sporten	☐	☐	☐
b	*matige inspanning* zoals het verplaatsen van een tafel, stofzuigen, fietsen.	☐	☐	☐
c	tillen of boodschappen dragen	☐	☐	☐
d	*een paar* trappen oplopen	☐	☐	☐
e	*één* trap oplopen	☐	☐	☐
f	buigen, knielen of bukken	☐	☐	☐
g	*meer dan een kilometer* lopen	☐	☐	☐
h	*een halve kilometer* lopen	☐	☐	☐
i	*honderd meter* lopen	☐	☐	☐

j	uzelf wassen of aankleden	☐	☐	☐

4 Had u, ten gevolge van uw **lichamelijke gezondheid**, *de afgelopen 4 weken* één van de volgende problemen bij uw werk of andere dagelijkse bezigheden?

		ja	nee
a	U heeft *minder tijd* kunnen besteden aan werk of andere bezigheden	☐	☐
b	U heeft *minder bereikt* dan u zou willen	☐	☐
c	U was beperkt in het *soort* werk of het soort bezigheden	☐	☐
d	U had moeite met het werk of andere bezigheden (het kostte u bijvoorbeeld extra inspanning)	☐	☐

5 Had u, ten gevolge van een **emotioneel probleem** (bijvoorbeeld doordat u zich depressief of angstig voelde), *de afgelopen 4 weken* één van de volgende problemen bij uw werk of andere dagelijkse bezigheden?

		ja	nee
a	U heeft *minder tijd* kunnen besteden aan werk of andere bezigheden	☐	☐
b	U heeft *minder bereikt* dan u zou willen	☐	☐
c	U heeft het werk of andere bezigheden niet zo zorgvuldig gedaan als u gewend bent	☐	☐

6 In hoeverre heeft uw **lichamelijke gezondheid** of hebben uw **emotionele problemen** u *de afgelopen 4 weken* belemmerd in uw normale sociale bezigheden met gezin, vrienden, buren of anderen?

- ☐ helemaal niet
- ☐ enigszins
- ☐ nogal
- ☐ veel
- ☐ heel erg veel

7 Hoeveel **pijn** had u *de afgelopen 4 weken*?

- ☐ geen
- ☐ heel licht
- ☐ licht
- ☐ nogal
- ☐ ernstig
- ☐ heel ernstig

8 In welke mate heeft **pijn** u *de afgelopen vier weken*

- ☐ helemaal niet

belemmerd bij uw normale werkzaamheden (zowel werk buitenshuis als huishoudelijk werk)?	☐ een klein beetje<br>☐ nogal<br>☐ veel<br>☐ heel erg veel

9 Deze vragen gaan over hoe u zich *de afgelopen 4 weken* heeft **gevoeld**. Wilt u bij elke vraag het antwoord omcirkelen dat het beste aansluit bij hoe u zich heeft gevoeld.

	Hoe vaak gedurende *de afgelopen 4 weken*:	voort-durend	meestal	vaak	soms	zelden	nooit
a	voelde u zich levenslustig?	☐	☐	☐	☐	☐	☐
b	voelde u zich erg zenuwachtig?	☐	☐	☐	☐	☐	☐
c	zat u zo erg in de put dat niets u kon opvrolijken?	☐	☐	☐	☐	☐	☐
d	voelde u zich kalm en rustig?	☐	☐	☐	☐	☐	☐
	Hoe vaak gedurende *de afgelopen 4 weken*:	voort-durend	meestal	vaak	soms	zelden	nooit
e	voelde u zich erg energiek?	☐	☐	☐	☐	☐	☐
f	voelde u zich neerslachtig en somber?	☐	☐	☐	☐	☐	☐
g	voelde u zich uitgeblust?	☐	☐	☐	☐	☐	☐
h	voelde u zich gelukkig?	☐	☐	☐	☐	☐	☐
i	voelde u zich moe?	☐	☐	☐	☐	☐	☐

10 *Hoe vaak* hebben uw **lichamelijke gezondheid** of **emotionele problemen** gedurende *de afgelopen 4 weken* uw sociale activiteiten (zoals bezoek aan vrienden of naaste familieleden) belemmerd?	☐ voortdurend<br>☐ meestal<br>☐ soms<br>☐ zelden<br>☐ nooit

11 Wilt u het antwoord kiezen dat het beste weergeeft hoe juist of onjuist u elk van de volgende uitspraken voor uzelf vindt.

	volkomen juist	groten-deels juist	weet ik niet	groten-deels onjuist	volkomen onjuist

a	Ik lijk gemakkelijker ziek te worden dan andere mensen	☐	☐	☐	☐	☐
b	Ik ben net zo gezond als andere mensen die ik ken	☐	☐	☐	☐	☐
c	Ik verwacht dat mijn gezondheid achteruit zal gaan	☐	☐	☐	☐	☐
d	Mijn gezondheid is uitstekend	☐	☐	☐	☐	☐

Categorie H

Curriculum vitae

Dr.John.Th.M. Plukker, surgeon

Born 05-12-1951
Paramaribo (Surinam)

Office Address:
Department of Surgical Oncology
University Medical Center Groningen
Hanzeplein 1, 9700 RB Groningen,The Netherlands
Phone:+31-3612317
Fax:+31-3614873
Email: j.th.plukker@chir.umcg.nl

Medical Education:

1969-1977 Medical School, University of Leiden, The Netherlands.

14 Jan 1977 Medical qualifying examination: RUL

CERTIFICATION

April 1984 Registration as General Surgeon

April 1986 Certification of Surgical Oncology

Clinical/Research fellow:

1971-1972 University of Surinam, Department of Pathology. (Head: Dr. F.Bellot)

1977-1978 Antoni van Leeuwenhoekhuis/The Netherlands Cancer Institute, Plesmanlaan 121, 1066 CX Amsterdam (Head: Prof.Dr. E.A. van Slooten) 1977-1978

1978 – 1984 Surgical Residency: Canisius Wilh. Hospital, Nijmegen (Head: Dr. H.J.Joosten) and Radboud University Hospital. (Head Prof. Dr.H.M.de Boer). Nijmegen, The Netherlands

1984-1985	Chef de Clinique; Canisius Wilhelmina Hospital Nijmegen
1985 may	**PhD.Thesis:**"The Akiyama procedure in the surgical management of esophageal cancer". University of Amsterdam. The Netherlands.
1985- 1987	Fellow of the Dutch Cancer Foundation at the Department of Surgical Oncology University Hospital Groningen, The Netherlands (Head: Prof Dr. J. Oldhoff)<br><br>Fellow at the National Cancer Center Tokyo, Japan (Head: Prof. Dr. K. Maruyama)<br><br>Fellow at the Instituto Nationale Dei Tumori, Milan, Italy (Head: Prof.Dr.U.Veronesi)
1987-1988	Staff member Department Of Surgery, Free University Hospital, Amsterdam, The Netherlands
1988- 2002	Assistant Professor of Surgery/Surg. Oncology. University Hospital Groningen, The Netherlands. Regional coordinator of the Comprehensive Cancer Center North Netherlands
2002- present	Associate Professor of Surgery/Surgical Oncology University Medical Center Groningen; particularly in the treatment of esophageal and gastric cancer and the treatment of endocrine tumors, including thyroid cancer.

Memberships:

1991- 1997	Medical Ethical Committee University Medical Center Groningen, University of Groningen.
1999- 2004	CKTO/NKB-KWF: Committee of Clinical Oncology National Cancer Foundation.
Present	Board member of the Comprehensive Cancer Center North Netherlands<br>Board member of the Northern Netherlands Oncology Centrum (NNOC)

Professional Membership of Societies

National:
Nederlandse Vereniging voor Heelkunde
Vereniging van Gastroenterologie
Vereniging van Chirurgische Oncologie

Abroad:
American Society of Clinical Oncology (ASCO)
Society of Surgical Oncology (SSO)
American Society of Colon and Rectal Surgeons (ASCRS)
American Association for Cancer Research (AACR)
International Gastric Cancer Association (IGCA)

The research group:

GE cancer, particularly Esophageal and Gastric cancer;

Dr. J.Th. Plukker is a research co-coordinator at the Department of Surgical Oncology, University Groningen. He is currently involved in the research and guidance of 5 PhD students working on grants from: The Netherlands Organisation for Health Research and Development Organisation (2x) and The University Medical Center Groningen (3x) of which he is the principal investigator).
His main research is clinical and basic science particularly on predictive factors in the treatment of esophageal cancer; improvements in staging and treatment of esophageal cancer. Furthermore, he is involved in several projects on this subject, including the national CROSS study on preoperative chemoradiation.

He is the principal investigator of:
1. The CIMPLE study (ZonMw project) to improve the staging of esophageal cancer patients (PhD fellow: C. Verhoef, start Febr 2005).
2. The DIVAPEC/Slokop study (ZonMw project) concerning the use and clinical value of PET in the staging of esophageal cancer patients had recently (2005) resulted in a PhD dissertation of two research fellows (H.L van Westreenen MD.PhD; PAM Heeren MD.PhD)

Several other projects in which he is/was the principal investigator;

1. IKN/CKVO trial 91-01: Adjuvant treatment of colon cancer patients with 5-FU/levamisol vs Leucovorin/5-FU/levamisole. PhD project of W. Bleeker MD.PhD.
2. KWF/NKB project 99-11: Molecular Characteristics as a Guidance for adjuvant treatment in colorectal carcinoma. (PhD fellows; J.L.Westra MD.PhD and W. Kelder MD)
3. The value of sentinel node procedure and molecular upstaging in colon cancer patients (PhD fellow: W.Kelder start January 2005)
4. Staging models in esophageal cancer: efficacy and cost (PhD fellow: L.Schreurs; start Jan. 2006)
5. MD PHD project: Optimal regional staging and impact on treatment results: Fellow B. Pultrum.
6. The D1 versus D2 trial in gastric cancer (regional coordinator)
7. The TME procedure in rectal cancer patients (regional IKN coordinator)

Oncological Endocrinology :Thyroid and parathyroid tumors.

Other principal research includes the treatment and detection of Thyroid cancer and parathyroid tumors:

1. MD.PhD RUG project on Medullary Thyroid Cancer.
   Treatment options and the value of tyrosine kinase inhibitors.
   (PhD fellow: JW de Groot MD. Dissertation May 2006)
2. Fine needle aspiration cytology and molecular biological markers.
   Value of TP53 and K-ras mutation in follicular lesions.
3. Treatment of Parathyroid lesions using minimal invasive methods and the value of qPTH and probe localisation. (PhD fellow: C.Jorna)

Publications:

John Plukker has been an (co)author on over 95 manuscripts, many of which appeared in highly rated journals such as NEJM, Lancet, JCO, Cancer and Ann of Surg Oncol.

A selection of papers:

1. Bonenkamp JJ, Songun J, Hermans J, Sasako M, Welvaart K, Plukker JTh, et al. Randomised comparison of morbidity after D1 and D2 dissection for gastric cancer in 996 Dutch patients. Lancet 1995; 345:745-48.
2. Bonenkamp JJ, Hermans J, Sasako M, van de Velde, Welvaart K, Songun I, Meyer S, Plukker JTM et al. Extended lymphnode dissection for gastric cancer. N. Engl J Med 1999;340:908-914.
3. Kole AC, Plukker JThM, Nieweg OE and Vaalburg W. Positron emission tomography for staging of oesophageal and gastroesophageal malignancy. Br.J.Cancer 1998;78:521-527.
4. Plukker JTh, Mulder NH, Sleijfer DTh, Grond J, Verschueren RCJ. Chemotherapy and surgery for locally advanced cancer of the cardia and fundus: phase II study with Methotrexate and 5-fluorouracil. Br J Surg 1991; 78:955-958.
5. Westra JL,Schaapveld M, Hollema H, de Boer JP, Kraak MM, de Jong D, ter Elst A, Mulder NH, Buys CH, Hofstra R, Plukker JThM. Determination of TP53 mutation is more relevant than microsatellite instability status for the prediction of disease-free survival in adjuvant-treated stage III colon cancer patients. JCO 2005;23:5623-43.
6. Haveman JW, Phan HT, Links TP,Jager PL,Plukker JTh. Implications of mediastinal uptake of 131I with regard to surgery in patients with differentiated thyroid carcinoma. Cancer 2005; 103:59-67.
7. van Westreenen HL, Westerterp M, Bossuyt PM, Sloof GW, van Lanschot JJ, Groen H, Plukker J ThM. Systematic review of the staging performance of 18F-fluorodeoxyglucose positron emission tomography in esophageal cancer. JCO 2004:22:3805-12
8. Haveman JW, van Tol KM, Rouwe CW, Piers DA, Plukker JTh. Surgical experience in children with differentiated thyroid carcinoma. Ann Surg Oncol 2003:10:15-20.
9. Bleeker WA, Mulder NH, Hermans J, Otter R, Plukker JTh. The addition of low-dose leucovorin to the combination of 5-fluorouracil- levamisole does not improve survival in the

adjuvant treatment of Dukes' C colon cancer. IKN Colon Trial Group. Ann Oncol. 2000:11:547-52.

10.de Groot JW, Links TP, Jager PL, Kahraman T, Plukker JTh. Impact of 18F-fluoro-2-deoxy-D-glucose positron emission tomography (FDG-PET) in patients with biochemical evidence of recurrent or residual medullary thyroid cancer. Ann Surg Oncol 2004:11: 786-94.

11. Westerterp M, van Westreenen HL, Reitsma JB, Hoekstra OS, Stoker J, Fockens P, Jager PL, Plukker JT, van Lanschot J, Sloof J. Esophageal cancer: CT, endoscopic US, and FDG PET for assessment of response to neoadjuvant therapy--systematic review. Radiology. 2005 Sep;236(3):841-51. Review.

12.de Groot JW, Sijmons RH, Links TP, Plukker JT, Hofstra RM. Medullary thyroid cancer in a patient with Hirschsprung disease with a C609Y germline RET-mutation. : J Pediatr Gastroenterol Nutr. 2005 Feb;40(2):226-9.

13.de Groot JW, Links TP, Jager PL, Kahraman T, Plukker JT. Impact of 18F-fluoro-2-deoxy-D-glucose positron emission tomography (FDG-PET) in patients with biochemical evidence of recurrent or residual medullary thyroid cancer. Ann Surg Oncol. 2004 Aug;11(8):786-94.

14.Plaza Menacho I, de Groot JW, Links T, Plukker J, Eggen BJ, Hofstra R. Re: "Inhibition of medullary thyroid carcinoma (MTC) cell proliferation and RET phosphorylation by tyrosine kinase inhibitors". Surgery. 2004 Feb;135(2):240-1; author reply 241.

15.Kahraman T, de Groot JW, Rouwe C, Hofstra RM, Links TP, Sijmons RH, Plukker JT. Acceptable age for prophylactic surgery in children with multiple endocrine neoplasia type 2a. Eur J Surg Oncol. 2003 May;29(4):331- 5.

Extra:

FDG-PET in de follow-up van medullair schildkliercarcinoom. de Groot JW, Links TP, Jager PL, Kahraman T, Plukker JT. Jaarvergadering Nederlandse Vereniging voor Nucleaire Geneeskunde, Groningen, Nederland, september 2003, **Voordracht**

Effects of STI571 on RET-mediated medullary thyroid carcinoma cell growth. de Groot JW, Plaza Menacho I, Schepers H, Drenth-Diephuis LJ, Plukker JT, Links TP, Eggen BJ, Hofstra RM. Annual

Meeting European Society of Clinical Investigation, Utrecht, Nederland, april 2004, **Voordracht**

Effects of imatinib mesylate on RET-mediated medullary thyroid carcinoma cell proliferation de Groot JW, Plaza Menacho I, Schepers H, Drenth-Diephuis LJ, Plukker JT, Links TP, Eggen BJ, Hofstra RM. THYROID CANCER FOUR, International Symposium, Styria, Oostenrijk, oktober 2004, **Voordracht**

Prophylactic thyroidectomy in patients carrying MEN2A RET-mutations. de Groot JW, Links TP, Plukker JT, Hofstra RM. THYROID CANCER FOUR, International Symposium, Styria, Oostenrijk, oktober 2004, **Voordracht**

Chirurgische behandeling van symptomatisch medullair schildkliercarcinoom. de Groot JW, Links TP, Sluiter WJ, Wolffenbuttel BH, Wiggers T, Plukker JT. Klinische Endocrinologie Dagen, Doorwerth, Nederland, februari 2005, **Voordracht**

Increased need for levothyroxine in thyroidectomized patients due to imatinib. de Groot JW, Zonnenberg BA, Plukker JT, van der Graaf WT, Links TP. Annual Meeting American Association for Cancer Research, Anaheim, USA, april 2005, **Poster**

Impact of 18F-fluoro-2-deoxy-D-glucose positron emission tomography (FDG-PET) in patients with biochemical evidence of recurrent or residual medullary thyroid cancer. de Groot JW, Links TP, Jager PL, Kahraman T, Plukker JT. Annual Meeting Society of Surgical Oncology, Los Angeles, USA, maart 2003, **Poster**

Curriculum Vitae

Barbara Louise van Leeuwen
Date of birth: 10 August 1971
Place of birth: Capelle aan den IJssel (The Netherlands)
Address: Van Goghstraat 37
9718 MR Groningen
The Netherlands
tel: 0031-50-5891968
email: barbara.van.leeuwen@hetnet.nl
barbara.van.leeuwen@znb.nl

Education

1983-1989: Highschool, St. Oelbert Gymnasium, Oosterhout, The Netherlands
1989-1997: Medicine, Erasmus University Rotterdam, The Netherlands
1995: United States Medical Licensing Examination (USMLE) Step 1
1996: United States Medical Licensing Examination (USMLE) Step 2

General Work Experience

1993-1994: Data collection in multi-center trial by Glaxo on the efficacy of ondansetron in postoperative nausea in Crawford Long Hospital, Atlanta GA, USA
1997-1999: House officer in surgery, University Medical Center Groningen, The Netherlands.

Surgical Training

2000-2003: Surgical resident University Medical Center Groningen
2003-July 2006 : Surgical resident Leeuwarden Medical Center, The Netherlands

Research

1993-1994: Study on the effect of therapeutic suggestions on postoperative recovery and morfine-use. This study was performed in the department of anesthaesia of Crawford W. Long Hospital of Emory University, Atlanta Georgia, USA
1997: Retrospective study on the outcome of the treatment of hyperthyroidism in the Royal Infirmary Edinburgh.
1998-2003: Phd on the effects of chemotherapy on the development of the growth plate and long bones, department of surgical oncology, University Medical Center Groningen. Thesis 14. May 2003 'The effects of chemotherapeutic agents on the growing skeleton'. Promotores Prof. Dr. H.J. Hoekstra (surgery) and Prof. Dr. W.A. Kamps (pediatrics)

Publications

- * Van Leeuwen BL, Hartel R, Jansen HW, Verkerke GJ, Veth RP, Kamps WA, Hoekstra HJ. Chemotherapy affects the pattern of failure after shear loading of the proximal tibial growth plate. *Archives of Orthopaedic and Trauma Surgery* 2004 Oct; 124 (8):503-6.
- * Van Leeuwen BL, Verkerke GJ, Hartel RM, Sluiter WJ, Kamps WA, Jansen HW, Hoekstra HJ. Chemotherapy decreases epiphyseal strength and increases bone fracture risk. *Clin Orthop*. 2003 Aug (413): 243-54.
- * Van Leeuwen BL, Hartel RM, Jansen HW, Kamps WA, Hoekstra HJ. The effect of chemotherapy on the morphology of the growthplate and metaphysis of the growing skeleton. *Eur J Surg Oncol*. 2003 Feb ;29 (1) :49-58.
- * Van Leeuwen BL, Pruim J, Gouw AS, van der Zee AG, Slooff MJ, de Jong KP. Liver metastasis as a first sign of fallopian tube carcinoma and the role of positron emission tomography in preoperative diagnosis. *Scand J Gastroenterol*.2002 Dec; 37 (12):1473-4.
- * Van Leeuwen BL, Kamps WA, Hartel RM, Veth RP, Sluiter WJ, Hoekstra HJ. Effect of single chemotherapeutic agents on the growing skeleton of the rat. *Ann Oncol*. 2000 Sep; 11(9):1121-6.
- * Van Leeuwen BL, Houwerzijl M, Hoekstra HJ. Educational tips in the treatment of malignant ulcerating tumours of the skin *Eur. J. Surg Oncol* 2000 Aug;26(5):506-508.
- * Van Leeuwen BL, Kamps WA, Jansen HW, Hoekstra HJ. The effect of chemothreapy on the growing skeleton.. *Cancer Treat Rev*. 2000 Oct;26(5):363-76.
- * Bringmann IM, Van Leeuwen BL, Hennemann G, Beckett GJ, Toft AD. Outcome of treatment of hyperthyroidism. *J Endocrinol Invest*. 1999 Apr;22(4):250-6.
- * Van Leeuwen BL, Houwerzijl M, Hoekstra HJ. Palliative care for cancer patients with refractory ulcerating malignant skin tumors and skin metastasis. *Ned Tijdschr Geneeskd*.1999 Mar 13;143(11):561-4.
- * Van der Laan WH, van Leeuwen BL, Sebel PS, Winograd E, Baumann P, Bonke B. Therapeutic suggestion has no effect on postoperative morphine requirements. *Anesth Analg*. 1996 Jan;82(1):148-52.

Categorie I

PROTOCOL SIGNATURE SHEET

Name	Signature	Date
For non-commercial research,<br>Head of Department:<br>Prof. Dr. T. Wiggers		14/10/06
Coordinating Investigator/Project leader/Principal Investigator:<br>Dr. B.L. van Leeuwen		13-10-06
Dr. J.P.E.N. Pierie		18-10-06
Dr. R. Vree		19/10/6

CURRICULUM VITAE

Personalia

Wiggers, Theodoor
geboren 2 april 1948 te Groningen

privé-adres:
Kamerlingheplein 6
9712 TR Groningen
050-3604279, 06-53347978
e-mail: wiggerst@wxs.nl
werkadres:
Academisch Ziekenhuis Groningen
Postbus 30.001
9700 RB Groningen
tel: 050-3612317 fax: 050-3614873
e-mail: t.wiggers@chir.umcg.nl

Loopbaan

juni 2005-heden
hoofd disciplinegroep chirurgie
Universitair Medisch Centrum Groningen

juni 2002-juni 2005
waarnemend hoofd chirurgie
Academisch Ziekenhuis Groningen

juni 2000- heden
hoogleraar chirurgische oncologie (hoofd chirurgische oncologie)
Academisch Ziekenhuis Groningen
hoofd: prof. dr. R. van Schilfgaarde (tot 1 juni 2002)

april 1998 – juni 2000
chirurg oncoloog (hoofd subafdeling chirurgische oncologie)
Academisch Ziekenhuis Rotterdam / Daniel den Hoed Kliniek
hoofd: prof. dr. J. Jeekel

januari 1990 – september 1998
clusterleider Snijdende Specialismen Dr Daniel den Hoed Kliniek

juni 1989 – juni 2000
consulent chirurg oncoloog Integraal Kankercentrum Rotterdam

juni 1984 – april 1998
chirurg oncoloog (vanaf 1990 hoofd afdeling chirurgische oncologie; tot juli 1994 deeltijd) Dr Daniel den Hoed Kliniek te Rotterdam

november 1986 – juli 1994
stafchirurg (deeltijd)
Zuiderziekenhuis te Rotterdam
hoofd: dr. G.A. Olthuis vanaf 1992 dr. K.J. Brouwer

juni 1984 – november 1986
chirurg (deeltijd)
Academisch Ziekenhuis Rotterdam / Dijkzigt
hoofd: prof. dr. H. van Houten

juni 1978 – juni 1984
arts-assistent heelkunde (in opleiding)
Ziekenhuis St. Annadal te Maastricht
hoofd: prof. dr. J.M. Greep
Stage plastische chirurgie
Ziekenhuis de Goddelijke Voorzienigheid te Sittard
hoofd: dr. T.J.B. de Goede
Stage radiotherapie
Radiotherapeutisch Instituut Limburg te Heerlen
hoofd: prof. dr. A.H. Keijser

maart 1976 – maart 1978
Arts
Bukoba Government Hospital te Bukoba in Tanzania

mei 1975 – oktober 1975
arts-assistent heelkunde
Nicolaas Tulp Ziekenhuis te Amstelveen
hoofd: dr. A. Nederveen

augustus 1974 – januari 1975
arts-assistent verloskunde en gynaecologie
Ziekenhuis Sint Joannes de Deo te Haarlem
hoofd: dr. L.C.L.M. Vasen

maart 1973 – september 1973 / mei 1974 – juli 1974
arts-assistent heelkunde
Sint Lucas Ziekenhuis te Amsterdam
hoofd: dr. J.M. Greep

september 1972 – november 1972
arts-assistent inwendige geneeskunde
Academisch Ziekenhuis Vrije Universiteit te Amsterdam
hoofd: prof. dr. E. Lopez Cardozo

Opleiding

1978 – 1984
Heelkunde in het Ziekenhuis Sint Annadal te Maastricht
inschrijving Medisch Specialisten Registratie Commissie: 1 juni 1984

1966 – 1974
Medicijnen aan de faculteit der Geneeskunde van de Vrije Universiteit te Amsterdam
Artsexamen: 5 april 1974

1960 – 1966
Gymnasium-ß aan het Christelijk Lyceum Buitenveldert te Amsterdam
Einddiploma: 6 juni 1966

Promotie

16 januari 1987
The no-touch isolation technique in colon cancer.
Report on a multicenter study with analyses of prognostic factors.
promotores: prof. dr. J.M. Greep en prof.dr. J. Jeekel

**Cursussen**	februari 1999<br>Auditortraining,<br>Nederlands Instituut voor Accreditatie van Ziekenhuizen (NIAZ)
	januari 1975 – maart 1975<br>National Course in Tropical Medicine and Hygiene,<br>Koninklijk Instituut voor de Tropen te Amsterdam
	25 juli 1973<br>Examen Educational Council for Foreign Medical Graduates (E.C.F.M.G.)
**Bestuursfuncties**	2003- heden<br>**Commissielid "Nacontrole bij kanker" van de Gezondheidsraad**
	2003- heden<br>**Commissielid "Effecten & Kosten" van ZONMw**
	2001-heden<br>**Bestuurslid " 'Behouden Huys"**
	2001- heden<br>**Editor Cochrane Colorectal Cancer Group**
	2001-heden<br>**Begeleidingscommissie Richtlijnen Orde van Medische Specialisten**
	1998 – 2000<br>**Oncologisch Beraad Academisch Ziekenhuis Rotterdam (AZR)**
	1998 – heden<br>**Executive Committee European Society for Surgical Oncology**<br>*Penningmeester 2004-*
	1997 – 2000<br>**Bestuur Integraal Kankercentrum Rotterdam (IKR)namens het AZR**
	1997 – 2001<br>**Landelijk Oncologisch Beraad**
	1996 – 1998<br>**Bestuur stafconvent AZR**
	1996 – heden<br>**Landelijke Werkgroep Gastro-Intestinale Tumoren**<br>*Voorzitter vanaf 1999*
	1996 – heden<br>**Concilium Chirurgicum**

1996 – 2000
Regionaal Netwerk Chirurg Oncologen IKR
Voorzitter van 1998- 2000

1996 – 2001
Redacteur Nederlands Tijdschrift voor Heelkunde

1996 – heden
Editorial Board van The European Journal of Surgical Oncology

1993 – 2000
Medische Adviesraad IKR
Plaatsvervangend voorzitter 1997- 2000

1993 – 1995
Bestuur Nederlandse Vereniging voor Heelkunde

1993 – 2000
Associate Editor van The European Journal of Surgery

1992 - 1993
Editorial Advisory Board van The European Journal of Surgery

1988 – 1992
Bestuur Klinisch Wetenschappelijke Staf Daniel den Hoed Kliniek
Voorzitter van 1988 – 1991 / vice voorzitter van 1991 – 1992

1988 – 2000
Protocollen commissie IKR

1988 – 1995
Bestuur Nederlandse Vereniging voor Chirurgische Oncologie
Voorzitter van 1992 – 1995

1986 – 1993
Commissie voor Klinisch Vergelijkend Onderzoek (CKVO) van de Nederlandse Kankerbestrijding
Voorzitter van 1990 – 1993

1968 – 1969
Bestuur S.S.R.A. in Amsterdam
Praeses

Opleider

2003- heden
Plaatsvervangend opleider heelkunde

2000- heden
Opleider vervolgopleiding chirurgische oncologie

Onderwijs

2000-2003
Coördinator co-assistentchap oncologie in cluster C van C 2000

1998 – 2000
Docent oncologie faculteit der Geneeskunde en Gezondheids-wetenschappen van de Erasmus Universiteit Rotterdam

1988 – 1998
Docent oncologie cursus Integraal Kankercentrum Rotterdam

1984 – 1998
Docent oncologie cursus Daniel den Hoed Kliniek

1982 – 1984
Lid planningsgroep en docent blok tumoren Rijksuniversiteit Limburg

Lidmaatschappen

Nederlandse Vereniging voor Heelkunde.
Nederlandse Vereniging voor Chirurgische Oncologie.
Nederlandse Vereniging voor Tropische Geneeskunde.
Nederlandse Vereniging voor Oncologie.
Nederlandse Vereniging voor Gastro-Intestinale Chirurgie.
Nederlandse Vereniging voor Gastroenterologie.
European Society of Surgical Oncology.
EORTC Gastro Intestinal Tract Cancer Cooperative Group.
American Society of Colon and Rectal Surgeons.
Fellow of the Royal College of Surgeons (Eng).
Society of Surgical Oncology
Society of Geriatric Oncology

Categorie K

ZonMw

Universitair Medisch Centrum Groningen
Raad van Bestuur
Drs. L.C. Bruggeman
Postbus 30.001
9700 RB GRONINGEN

Laan van Nieuw Oost Indië 334
2593 CE Den Haag
Postbus 93245
2509 AE Den Haag
T 070 349 5111
F 070 349 5100
info@zonmw.nl
www.zonmw.nl

contactpersoon
Carolien Bouma
T 070 349 5260
F 070 349 5389
doelmatigheidsonderzoek@zonmw.nl

dossiernummer 945-07-516
ons kenmerk 2006/15800/ZONMW
datum 06 oktober 2006
pagina 1 van 2

onderwerp honorering subsidieaanvraag, projectnummer 945-07-516

Geachte heer Bruggeman,

In onze brief van 29 juni hebben wij u medegedeeld dat ZonMw uw subsidieaanvraag met de titel *A controlled trial of geriatric liaison intervention in frail surgical oncology patients (80-007022-98-07516)* zou honoreren nadat u ons de startdatum en het patiënteninclusieschema zou opsturen. Hieraan heeft u voldaan waardoor ik u met genoegen meedeel dat uw subsidieaanvraag is gehonoreerd. Bij verdere correspondentie en vragen aan ZonMw over het project vragen wij u uw projectnummer (945-07-516) te vermelden.

Financiering
De financiële bijdrage van ZonMw voor uw project bedraagt in totaal maximaal € 351.984,00 voor de duur van maximaal 36 maanden. Dit bedrag is inclusief eventueel verschuldigde BTW. De financiële bijdrage van ZonMw is ten behoeve van de inclusie van 351 patiënten en leidend tot 264 volledig ingevulde Case Report Forms (CRF's) van evalueerbare patiënten à € 1333,27 per CRF. De startdatum van uw project is 01-01-2007 en de looptijd is 36 maanden.

Aan de financiering zijn voorwaarden verbonden. Hiervoor verwijs ik u naar de bijgevoegde subsidievoorwaarden ZonMw, 1 juli 2004 en de aanvullende voorwaarden voor het programma DoelmatigheidsOnderzoek.

Het is belangrijk dat u ZonMw zo spoedig mogelijk, doch uiterlijk binnen 4 weken schriftelijk o.v.v. toekenning projectnummer 945-07-516 laat weten:

- Of u instemt met de voorwaarden die van toepassing zijn op de toekenning van de financiële bijdrage;
- De bank –en referentiegegevens die van toepassing zijn bij de betalingen van de subsidie.

Pas na uw instemming met de voorwaarden én na de daadwerkelijke start van uw project, zal ZonMw het voorschot voor het eerste projectjaar verstrekken. Over de hoogte van het bedrag en de betaaltermijnen zullen wij u nader informeren.

Heeft u naar aanleiding van deze brief vragen, neemt u dan gerust per e-mail of eventueel telefonisch contact op met de contactpersoon die in het briefhoofd vermeld staat. Ik verzoek u vriendelijk in uw communicatie met ZonMw steeds het projectnummer te vermelden.

Ik wens u veel succes met de uitvoering van uw project.

Met vriendelijke groet,
namens het bestuur,

Henk J. Smid
directeur

Kopie
Prof. dr. J.P.J. Slaets, projectleider en penvoerder
Dr. H. Groen, contactpersoon DoelmatigheidsOnderzoek
Jac's den Boer & Vink BV controller
F&C, ZonMw

Bijlagen
Subsidievoorwaarden ZonMw, 1 juli 2004
Aanvullende subsidievoorwaarden programma DoelmatigheidsOnderzoek

Wilt u uw mening geven, klagen of bezwaar maken?

1. U kunt (liefst schriftelijk) uw mening geven. ZonMw vat dit op als een signaal en behandelt uw opmerkingen zorgvuldig en serieus.
2. U kunt schriftelijk een klacht indienen. ZonMw heeft een speciale klachtenprocedure. De directie van ZonMw behandelt iedere schriftelijk ingediende klacht.
3. U kunt bezwaar maken tegen een beschikking. Stuurt u dan binnen zes weken na de dag waarop het besluit bekend is gemaakt een bezwaarschrift aan de Commissie beroep- en bezwaarschriften ZonMw, p/a Postbus 93 138, 2509 AC Den Haag.

ZonMw adviseert u de folder Signaleren, klagen en bezwaar maken te lezen (www.zonmw.nl). Deze kunt u gratis opvragen via ons algemeen telefoonnummer (070) 349 51 11 of per e-mail: info@zonmw.nl.

Het is raadzaam eerst contact op te nemen met de programmamedewerker van ZonMw (de contactpersoon). Niet altijd hoeft u voor het 'zware' middel van het bezwaarschrift te kiezen. In de praktijk blijken problemen en ontevredenheid rond de ZonMw-procedure vaak naar tevredenheid opgelost te worden na (telefonisch) contact met de programmamedewerker.
